# Multifaceted functions of STING in human health and disease: from molecular mechanism to targeted strategy

**DOI:** 10.1038/s41392-022-01252-z

**Published:** 2022-12-23

**Authors:** Zili Zhang, Haifeng Zhou, Xiaohu Ouyang, Yalan Dong, Alexey Sarapultsev, Shanshan Luo, Desheng Hu

**Affiliations:** 1 Department of Integrated Traditional Chinese and Western Medicine, Union Hospital, Tongji Medical College, Huazhong University of Science and Technology, 430022 Wuhan, China; 2 Institute of Immunology and Physiology, Ural Branch of the Russian Academy of Science, 620049 Ekaterinburg, Russia; 3 Institute of Hematology, Union Hospital, Tongji Medical College, Huazhong University of Science and Technology, Wuhan, 430022 China; 4 Key Laboratory of Biological Targeted Therapy, The Ministry of Education, 430022 Wuhan, China; 5 Clinical Research Center of Cancer Immunotherapy, 430022 Hubei Wuhan, China

**Keywords:** Structural biology, Immunology

## Abstract

Since the discovery of Stimulator of Interferon Genes (STING) as an important pivot for cytosolic DNA sensation and interferon (IFN) induction, intensive efforts have been endeavored to clarify the molecular mechanism of its activation, its physiological function as a ubiquitously expressed protein, and to explore its potential as a therapeutic target in a wide range of immune-related diseases. With its orthodox ligand 2’3’-cyclic GMP–AMP (2’3’-cGAMP) and the upstream sensor 2’3’-cGAMP synthase (cGAS) to be found, STING acquires its central functionality in the best-studied signaling cascade, namely the cGAS–STING–IFN pathway. However, recently updated research through structural research, genetic screening, and biochemical assay greatly extends the current knowledge of STING biology. A second ligand pocket was recently discovered in the transmembrane domain for a synthetic agonist. On its downstream outputs, accumulating studies sketch primordial and multifaceted roles of STING beyond its cytokine-inducing function, such as autophagy, cell death, metabolic modulation, endoplasmic reticulum (ER) stress, and RNA virus restriction. Furthermore, with the expansion of the STING interactome, the details of STING trafficking also get clearer. After retrospecting the brief history of viral interference and the milestone events since the discovery of STING, we present a vivid panorama of STING biology taking into account the details of the biochemical assay and structural information, especially its versatile outputs and functions beyond IFN induction. We also summarize the roles of STING in the pathogenesis of various diseases and highlight the development of small-molecular compounds targeting STING for disease treatment in combination with the latest research. Finally, we discuss the open questions imperative to answer.

## Introduction

Innate immune responses are the ‘frontline troops’ that provide an immediate and nonspecific response to cellular stresses or pathogenic invasions, which are intricately bridged with adaptive immunity to jointly maintain immune homeostasis. However, in the history of immunology, the understanding of innate immunity comes to light posterior to that of adaptive immunity. In the last decade of the 20th century, the progress on the Toll-like receptors (TLRs) on the cell membrane greatly expanded our understanding of innate immunity recognition and validated the pathogen-associated molecular pattern (PAMP) theory. However, how non-self-signal in the cytoplasm, including nucleic acid, is recognized remains elusive. Stimulator of interferon genes (STING) was found in 2008 as the key adaptor in innate immunity for the cytosolic recognition of both pathogen-derived and self- DNA.^1–4^

Over a decade of research, the most recognized function of STING is embodied in the cyclic GMP–AMP synthase (cGAS)–STING–Interferon (IFN) pathway. cGAS senses aberrant double-stranded DNA (dsDNA) exposure in the cytosol and synthesizes 2′3′ cyclic GMP–AMP (2’3’-cGAMP or cGAMP),^5,6^ which, as the second message, binds to and potently activates the STING located in the endoplasmic reticulum (ER). STING then takes a conformational change and translocates from ER to the perinuclear compartment, where it forms a speck-like structure and recruits TANK-binding kinase 1 (TBK1) to produce highly ordered consecutive phosphorylation.^7,8^ The key substrate is interferon regulatory factor 3 (IRF3), which is phosphorylated to be a dimer and enters the nucleus to initiate type I IFN production.^8^

In evolutionary perspective, the origin of the STING homologue can be found in bacteria, while the best-studied type I IFNs as their output only emerge in vertebrates.^9^ It is indicated that STING may inherit more primordial functions, since STING is a germline-coded and ubiquitously expressed protein in nonhematopoietic cells, such as myocyte, neuron, adipocyte, and islet cells, etc.^2,10^ To support this deduction, recent studies revealed that STING activation also initiates NF-κB activation,^1^ cell death,^11,12^ endoplasmic reticulum (ER) stress,^11^ autophagy,^13,14^ translation inhibition,^15^ DNA damage response, and metabolic reprogramming.^16^ However, their regulations and underlying mechanisms are less known.

A recent structural study pointed out the existence of a second ligand pocket in the STING transmembrane domain, which was reminiscent of the discovery of cGAMP about 10 years ago and revealed brand-new details of STING activation. Furthermore, the STING phase separation was also reported.^17^ However, it is also necessary to further investigate the detailed biochemical mechanism of STING translocation, polymerization, and substrate recruitment.

In this Review, our objective is to summarize the versatile outputs of STING and elucidate the molecular mechanism of STING activation with reference to its structural data. In addition, STING-related diseases and drugs targeting STING for treatment are also presented in light of the most recent findings. Finally, we discuss the questions imperative to be answered.

## A brief history of innate immune research before the discovery of STING

In early 1937, the phenomenon that monkeys infected by one virus were protected from one another virus in an antibody-independent way was then named virus interference.^18^ Twenty years later in 1957, the active substance responsible for conferring this resistance was discovered to be IFN.^19^ IFN induction can also be induced by heated virus or a nucleic acid derived from cells not infected with viruses, implying that foreign nucleic acid is the stimulus.^18^

The transcriptional regulation of cytokines was not appreciated until the discovery of NF-κB in the late 1980s.^20^ And specialized transcription factors for IFN induction were discovered to be the interferon transcription factors (IRFs) family.^21,22^ IFN induction mediated by the TBK1–IRF3 axis and NF-κB activation were deemed as two hallmark events of viral infection.^23^ The open question is to probe the upstream sensors for nucleic acid. TLRs are located on the cell membrane and in a subset of immune cells, which cannot explain why all nucleated cells are responsible for viral infection with IFN production. These clues indicate a more ubiquitously expressed sensor of nucleic acid existing in the cytosol.

**Fig. 1:**
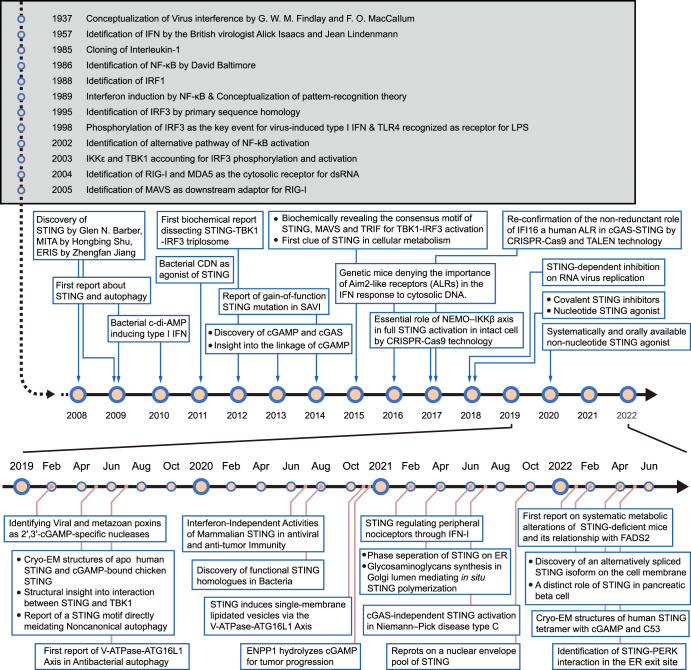
Timeline depicting the brief history of interferon discovery and key events about STING biology since its identification in 2008. ATG16L1, autophagy-related 16 like 1; CDN cyclic dinucleotides, cGAMP cyclic GMP–AMP, CRISPR/Cas9 clustered regularly interspaced short palindromic repeats/CRISPR-associated protein 9, ENPP1 ectonucleotide pyrophosphatase/phosphodiesterase 1, ERIS endoplasmic reticulum IFN stimulator, IFN interferon, IRF1 interferon regulatory factor 1, IRF3 interferon regulatory factor 3, LPS lipopolysaccharide, MITA mediator of IRF3 activation, PERK PKR-like endoplasmic reticulum kinase, STING stimulator of interferon genes, TALEN transcription activator-like effector nucleases, TBK1 TANK-binding kinase 1, TLR4 Toll-like receptor 4, SAVI STING-associated vasculopathy with onset in infancy

The research on the mechanism of cytosolic RNA detection then took the lead. In 2004, retinoic acid-inducible gene I (RIG-I) and melanoma differentiation-associated gene 5 (MDA5) were found to be the cytosolic dsRNA sensor^24,25^ and in the following 2005 its downstream adaptor mitochondrial antiviral signaling (MAVS) (also known as IPS-1/VISA/CARDIF) was described,^26–29^ which now constitutes the RIG-I–MAVS pathway responsible for RNA detection. On the contrary, the sensor for cytosolic DNA is long missing. In 2006, two groups reported the induction of type I IFN when double-stranded DNA (dsDNA) was introduced into the cytoplasm by transfection.^30,31^ Although these two studies did not find the dsDNA detector, they reached a consensus on the essential role of IRF3 and the independence of TLRs. Eventually, in 2008, STING was discovered as the adaptor for cytosolic DNA signaling (Fig. 1).

## The inputs of STING activation

### The discovery of STING, cGAMP, and cGAS

STING (also known as MPYS, MITA, ERIS, and TMEM173) was discovered in succession by four independent groups.^1–3,10^ Actually, the first group identified STING (MPYS in that paper) through the mass spectrum as an MHC-II-associated membrane adaptor, which transduced signals from the cognate MHC-II–TCR interaction and led to Fas-independent antigen-presenting cell (APC) death via ERK activation.^10^ The other three groups all used luciferase reporter-based cDNA expression screening and identified STING as the key adaptor to mount type I IFN induction upon DNA virus infection or dsDNA transfection.

This effect exists in not only immune cells but also stromal cells, as supported by the expression of STING in a wide range of tissues. Murine and human STING share 68% sequence identity at the amino acid level.^32^ STING has putative orthologs in diverse species along evolution but is not homogeneous to any known or predicted proteins. STING is believed to be the smallest transmembrane protein whose near-atomic structure has been resolved by cryo-EM until now.^33^ Human STING is a 379 amino acid protein with a calculated molecular weight of 42 kDa. STING activation results in its translocation from the ER in a dispersed distribution to the non-ER perinuclear compartment and assembly into punctate structures with TBK1 colocation, which is deemed the hallmark of STING activation under a microscope.

How STING senses dsDNA is still elusive at that time. The cyclic dinucleotides (CDN) c-di-GMP and c-di-AMP from bacteria induced a transcriptional profile similar to that of the cytosolic dsDNA.^34,35^ STING was validated to be a direct sensor of c-di-GMP.^36,37^ However, such PAMP is absent in viruses. This gap was bridged by the work of Zhijian James. Chen and his colleagues in 2013. They identified cGAMP and its synthase using biochemical purification and quantitative mass spectrometry.^5,6^ In detail, they did neither stick to the STING direct interactome for searching nor regard the DNA-binding domains as the gold criteria for the candidate DNA sensor. Instead, they ingeniously divided STING from the putative upstream activator and identified that this activator in cell extracts is heat, benzonase, and proteinase K resistant, and is cell-permeable in PFO-treated reporter cells. This strategy excluded the STING activator as a protein, DNA, or RNA and largely narrowed the scope. After the identification of cGAMP, they focused on cytosolic extracts with cGAMP synthesizing activity. The cGAS was finally identified in fractions subjected to three independent purification routes by quantitative mass spectrometry.

Subsequent studies identified that cGAS-synthesized cGAMP had a unique 2’–5’ phosphodiester bond and differed from bacteria-derived cyclic dinucleotides, making it also the first discovered CDN in mammalian cells.^38,39^ 2’3’-cGAMP has a higher binding affinity and activation potency to STING than c-di-AMP and c-di-GMP.^40^

The discovery of cGAS and cGAMP greatly compensates for the gap in dsDNA recognition, mirroring the RIG-I/MDA5–MAVS pathway in RNA recognition. And the importance of cGAS for the sensation of cytosolic dsDNA was soon demonstrated in transgenetic mice knocked out of cGAS.^41^

### Other putative DNA sensors and STING activators

Besides cGAS, several proteins have been proposed to function as DNA ‘sensors’, such as ZBP1, IFI16, DDX41, DNA-PK, MRE11, PQBP1, and ALR.^42–47^ Most of them lack conclusive experimental evidence and have been reviewed elsewhere.^48^ Among them, IFI16 is the most controversial, which belongs to the AIM2-like receptors (ALRs) gene family. In 2016, Gray et al. generated primary IFI16-depleted mice and human fibroblasts by CRISPR-Cas9 and revealed that IFI16 was dispensable for IFN type I production in response to transfected DNA ligands, DNA virus infection, and lentivirus infection.^49^ Dramatically, two subsequent studies reclaimed the essential role of IFI16 in cytosolic DNA sensation and IFN induction using gene editing technology.^50,51^ The controversy about IFI16 may be involved in its cell-specific function and is pending further investigation. It has been proposed that in etoposide-induced DNA damage, ataxia telangiectasia mutated (ATM) and IFI16 can activate STING by an alternative STING signaling complex and independently of cGAS.^52^

### Structural insight into two pockets of STING

**Fig. 2:**
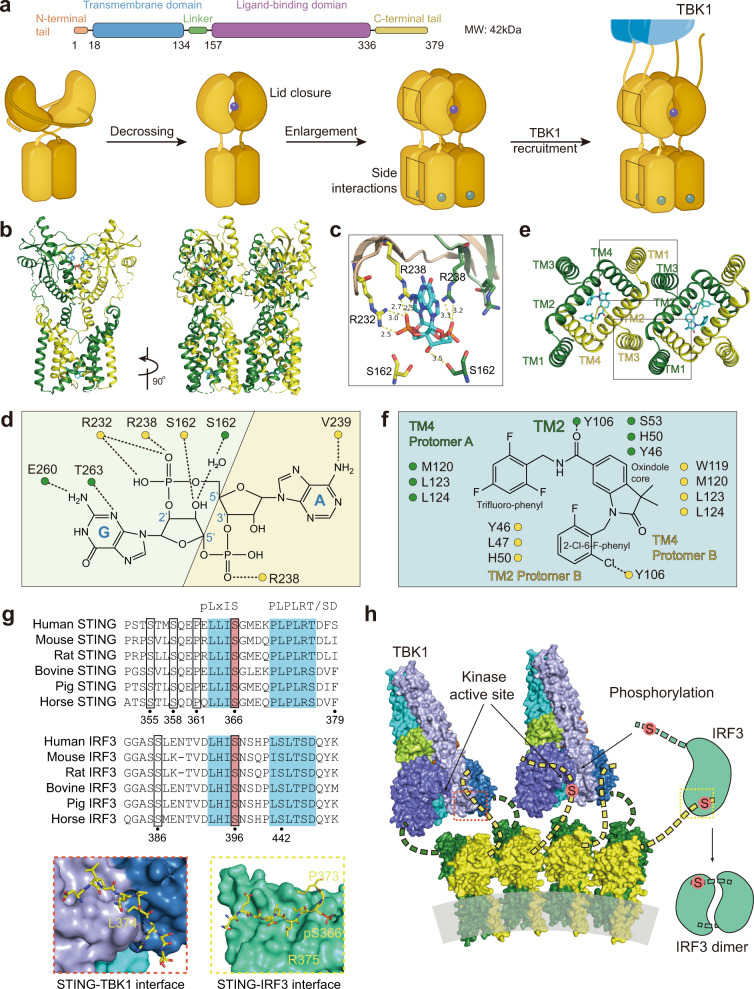
Molecular insight into STING activation. **a** Upper panel, the schematic domain representation of human STING (molecular weight, 42 kDa); bottom panel, conformational changes of STING activation. In steady-state, the transmembrane helix of two protomers forms a domain-swapped architecture. The STING LBD dimer presents a V shape, with a deep cleft between the two protomers to accommodate the CDNs, as the first pocket. (Graphic modified from Fig. 4 of ref. ^33^ and Extended Data Fig. 7 of ref. ^40^). **b** Cartoon representation of the structure in two orthogonal side views of activated STING tetramer with 2’3’-cGAMP and C53. (STING tetramer bound to both cGAMP and C53, PDB ID: 7SII). **c** Insight into the 2’-3-cGAMP-binding pocket. Residues offer key interaction with 2’3’-cGAMP and C53. The guanidinium groups of R238 on the lid sheet hang down into the core of LBD and forms direct interaction with the bottom backbone phosphates. The phosphate of 2’-5’ phosphodiester peripherally contacts with the R232 on one side. The free 3’-OH of guanosine forms a direct or water-mediated hydrogen bond to two Ser162 residues from the lower part of the pocket, whereas 2’-OH of adenosine is free of interaction. The guanine base directly interacts with the side groups of Glu260 and Thr263, while the adenosine forms only interaction with the main-chain carbonyl oxygen of Val239. (cGAMP bound human STING CTD structure, PDB ID: 4KSY). **d** Two-dimensional diagram of the interactions between STING ligand binding domain and 2’3’-cGAMP in the cytosolic side. **e** Two four-helix bundles are connected at the ER or Golgi luminal side by the N-terminal residues to maintain the TMD–TMD interaction between STING dimers. (STING tetramer bound to both cGAMP and C53, PDB ID: 7SII). **f** Two-dimensional diagram of the interactions between STING transmembrane domain and C53. **g** The C-terminal tail (CTT) of STING contains two conserved motifs for TBK1 and IRF3 binding, PLPLRT/SD motif and pLxIS motif (p, hydrophilic; x, nonaromatic) (up). The IRF3 CTT harbors a similar conserved cLxIS (c, charged residue) consensus motif. **h** Model of TBK1 activation and STING and IRF3 phosphorylation upon STING oligomerization. TBK1 sits above and binds to the CTT of STING dimer, but phosphorylates the CTT of an adjacent STING dimer. IRF-3 contains the binding surface for both the pLxIS motif of STING to mediate its recruitment and phosphorylation by TBK1 and also a pLxIS motif of its own to mediate its dimerization. Close-up views of the STING–TBK1 interface and STING–IRF3 interface are zoomed in and presented in red and yellow dashed boxes, respectively. (TBK1–STING tail complex, PDB: 6NT9; Phosphated STING tail–IRF3 complex, PDB: 5JEJ; cGAMP-bound chicken STING tetramer, PDB: 6NT8). All structural figures were generated with PyMOL (https://www.pymol.org). cGAMP cyclic GMP–AMP, IFN interferon, IRF3 interferon regulatory factor 3, STING stimulator of interferon genes, TBK1 TANK-binding kinase 1

STING can be divided into three main domains: the transmembrane domain (TMD), the cytoplasmic ligand-binding domain (LBD), and the C-terminal tail (CTT) (Fig. 2a). STING exists mainly as a symmetrical dimer, with the LBD opening toward the cytoplasm.^53–57^ The LBD of STING accommodates 2’3’-cGAMP. In the most recently discovered cryo-EM of human STING tetramer, a synthetic human STING agonist C53 is docked into the STING transmembrane domain, which is coined as the second pocket of STING.^58^ Both 2’3’-cGAMP and C53 induced key conformational changes for STING activation. The 2’3’-cGAMP ligation induces an inward rotation of two protomers in relation to the 2’3’-cGAMP-binding site, and the formation of the four-stranded antiparallel β sheet cap associated with the ‘open’ to ‘closed’ transition, which is highly disordered in the apo structure. Another important feature is a 180° rotation of the LBD relative to the TMD unwinding the intradimer crossover, which is only visible in the near-full-length STING. The STING then oligomerizes through side-by-side packing (Fig. 2a, b).

All these conformational changes are believed to be driven by extensive interactions between 2’3’-cGAMP and the STING LBD pocket, which propagate intermolecular force outward to the surface of the STING. Several residues offer a key interaction with 2’3’-cGAMP, including R238, R232, Ser162, Glu260, and Thr263. Most recent research validates the primordial origin of STING and its fundamental immune role in bacteria. A critical feature absent in bacterial STING receptors is additional arginine-specific contacts to the phosphodiester backbone. Human STING R232 side chain contact, known to be critical for high-affinity interactions with 2′,3′-cGAMP, is conserved throughout metazoan STING^9^ (Fig. 2c, d). Of note, four major STING SNPs exist in the human population: R232H (13.7%), R293Q (20.4%), G230A-R293Q (AQ, 5.2%), and R71H-G230A-R293Q (HAQ, 1.5%). It is reported that these all four major SNPs could recognize 2’3’ cGAMP, but responded differentially to bacterial cyclic dinucleotides.^59^

And C53 ligation induced substantial sideways expansion of the transmembrane (TM) helices, which then forms two four-helix bundles in the TMD interface to stabilize the oligomer.^58^ The TMD interaction greatly contributes to the side-by-side packing of the STING by hydrophobic residues, while the cytosolic LBD contact between the two STING dimers seems to be weak and small. STING dimers associate more closely on the luminal side than on the cytosolic side, leading to the overall curvature of high-order STING oligomers (Fig. 2e, f).

It is noteworthy that 2’3’-cGAMP is negatively charged, and hydrophilic but membrane impermeable, while C53 is mostly hydrophobic.^58^ It remains unknown whether an endogenous ligand exists as C53 did. Sulfated glycosaminoglycans (sGAGs) have been reported to mediate STING polymerization by targeting the luminal loop of STING,^60^ quite matching the mechanism of C53. The exclusive synthesis of sGAGs in the Golgi lumen also underlies why STING requires translocation to the Golgi apparatus. Negative sulfate groups in sGASs played a key role in mediating the multivalent electrostatic interactions with STING.^60^ The interaction between sGASs and STING is warranted by further structural study. Similarly, a free glycan, Manβ1-4GlcNAc disaccharide, stimulates a broad immune response in vitro, which is in part dependent on the STING–TBK1 pathway.^61^

### Alternative activation of STING

STING activation is a promising strategy to fight against tumors. Several strategies develop in parallel to design the STING agonist. Outside these two pockets, a polyvalent and pH-sensitive STING agonist is reported to induce STING polymerization by binding to negatively charged residues (E296, D297) on the α3 helix of the ligand-binding domain (LBD).^62^ Manganese (Mn) was also found to act as an adjuvant to boost STING activation.^63,64^

## The diverse outputs of STING

The IFN induction mediated by STING–TBK1–IRF3 is the best-known and well-studied output of STING activation. However, this function only emerges in the mammalian cell from the perspective of evolution. NF-κB activation is another important output of STING and has a more ancient existence in invertebrates.^65,66^ Though NF-κB is more ancient than IRF3 activation, both outputs exerted an effect through cytokine induction and were activated in a relatively late stage of STING activation, before which STING had to be translocated across a long secretory path. Recently, accumulating studies indicate that STING activation yields more versatile outputs beyond cytokine induction, such as autophagy, ER stress, metabolic reprogramming, and translation inhibition, some of which take effect in a relative upstream timeline of STING activation. Their mechanisms are also less clear. Here, we summarize the research progress on them.

### Structural insight into STING–TBK1–IRF3 signalosome

The molecular mechanism of the interaction between STING, TBK1, and IRF3 is resolved by both biochemical assay and structural study. The STING CTT contains two important motifs for the binding of TBK1 and IRF3, respectively, namely the PLPLRT/SD motif^67,68^ and the pLxIS motif (p, hydrophilic; x, nonaromatic)^8^ (Fig. 2g). The PLPLRT/SD motif, located downstream of the pLxIS motif in the STING CTT, inserts into a groove between the kinase domain of a TBK1 subunit and the scaffold and dimerization domain (SDD) of the second subunit in the same TBK1 dimer, where the residue Leu374 in STING makes a significant contribution to stabilizing the interaction.

The STING oligomer platform brings together multiple TBK1 dimers and contributes to the trans-autophosphorylation of TBK1 in proximity marked by Ser172 phosphorylation. The catalytically activated TBK1 molecules then phosphorylate the Ser366 residue within the pLxIS motif of an adjacent STING dimer, but not of the STING dimer to which it binds. Upon phosphorylation, this motif serves as a docking site to recruit IRF3. In detail, IRF3 harbors a similar conserved consensus motif cLxIS (c, charged residue) in the C terminal, which can be similarly phosphorylated by TBK1 as the STING pLxIS motif did. On its N terminal is a positively charged surface, which can accommodate the phosphorylated form of both the pLxIS motif in STING and the cLxIS of another IRF3. In this way, IRF3 is recruited onto the STING oligomer by binding to the phosphorylated STING CTT and then forms an IRF3 dimer (Fig. 2h). This ‘licensing’ mechanism mediated by the pLxIS motif is also shared by IFN-producing adapters, such as Toll/interleukin-1 receptor domain-containing adapter protein (TRIF), MAVS^8^ and TLR adaptor interacting with endo-lysosomal SLC15A4 (TASL).^69^

### Serine phosphorylation in the STING–TBK1–IRF3 signalosome

Most studies exploited the phosphorylation of IRF3 Ser396 and STING Ser366 as markers for its activation. Actually, there are serines near the p/cLxIS motif that can also be phosphorylated. The human STING S358A mutant (corresponding to the murine STING S357A mutant) also presented a diminished ability to activate IRF3 or impair IFN-β reporter activation.^70^ The kinase for its phosphorylation is unclear. As the IRF3 activation mechanism was first reported in 1998, two serine residues S396 and S386 of IRF3 were independently reported to play a more important role in IRF3 activation, which was a historical dispute.^22,71^ However, a recent structural and biochemical study emphasized the importance of S383 in maintaining IRF3 dimer and IFN induction.^70,72^ However, it is not clear whether TBK1 is responsive to the phosphorylation of all these residues.

Notably, all members of the AKT kinase family (also named protein kinase B/PKB) family were recently found to participate in STING–IRF3 activation but elicited contrasting effects. AKT3 can increase IRF3 activation by phosphorylating the S385 residue.^73^ HER2 strongly associates with STING and recruits AKT1 to directly phosphorylate TBK1, which prevented the association of TBK1-STING and TBK1 K63-linked ubiquitination.^74^ AKT2 negatively regulates I-IFN production by phosphorylating IRF3 on Thr207 and attenuating the nuclear translocation of IRF3. The ALK–EGFR–AKT axis promotes STING activation.^75^ And for the cGAMP-unresponsible spontaneous tumor model, combined usage of the AKT inhibitor can potentiate the antitumor effect induced by cGAMP, while the mechanism was unclear.^76^ Mutant p53 can bind to TBK1 and prevents the formation of a trimeric TBK1–STING–IRF3 complex.^77^

### NF-κB activation in STING-containing SMOCs

The promoter region of IFN contains redundant positive regulatory domains (PRDs) that were inclusively modulated by transcriptional factors including IRFs, NF-κB and AP-1.^78^ Though IRFs specify IFN induction, NF-κB aids in IFN production on the transcriptional level, especially in the early phase when IRF3 activation is low.^79^ Recent study also confirms a fundamental and contributing role of NF-kB activation for some STING biological functions, which are less impaired when IFN induction is selectively dampened.^80^ However, the mechanism to elicit NF-κB activation by STING at a molecular resolution is much less clear.

In contrast to the consensus on the essential role of STING CTT in mediating IRF3 activation, whether such a tail is dispensable for NF-kB activation is currently controversial. On the one hand, it has been shown that the STING CTT, which is the docking site for TBK1 recruitment, is necessary for NF-κB activation.^81,82^ On the other hand, however, the STING homologue of Drosophila (dSTING) lacks a CTT motif but can still initiate NF-κB signaling to exert antiviral response, even when expressed in human 293T cells.^66^ The construction of a STING knockout cell with the CTT deleted form of STING did not impair the NF-κB activation.^83,84^ But it should be interpreted with caution that the absence of CTT did not mean that TBK1 is not involved in this process, because genetic evidence from the TBK1 knockout cell confirmed the importance of TBK1 in NF-κB activation.^79,85^ Thus, TBK1 may be recruited to STING for NF-κB in an indirect way, which is different from the STING–TBK1–IRF3 triplosome.

**Fig. 3:**
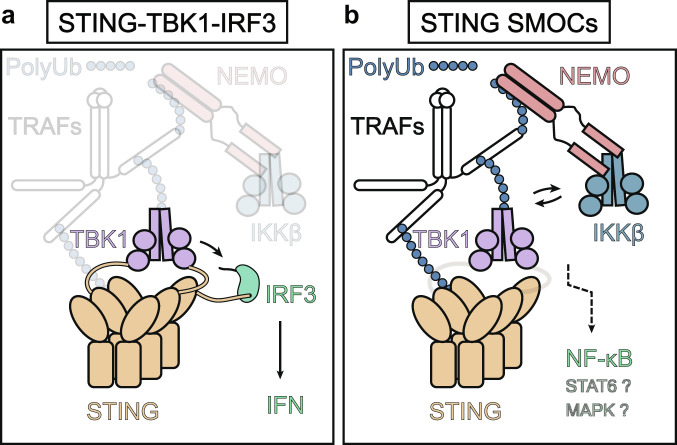
A proposed STING SMOCs model reconciling the TBK1-IRF3 and NF-κB activation. **a** STING–TBK1–IRF3 complex activates IRF3 and induces IFN production in a STING C-terminal tail-dependent way. **b** Activated STING polymerizes and recruits essential adaptors like TRAFs, NEMO, and IKKβ, beyond the STING–TBK1–IRF3 complex. Ubiquitination chains are covalently linked to various components of these SMOCs and stabilize the complex. A positive feedback loop exits between TBK1 and IKKβ to assure full activation of NF-κB, which may not depend on the STING C-terminal tail but require TBK1. Enigmatic mechanisms of STAT6 and MAPKs activation could also originate in this complex. cGAMP cyclic GMP–AMP, IFN interferon, IKKβ inhibitor of NF-κB kinase beta, IRF3 interferon regulatory factor 3, NEMO NF-κB essential modifier, PolyUb polyubiquitin chain, STING stimulator of interferon genes, SMOCs supramolecular organizing centres, TBK1 TANK-binding kinase 1, TRAFs tumor necrosis factor receptor-associated factors

It was postulated that STING may activate NF-kB in the framework of supramolecular organizing centers (SMOCs),^86^ which is a concept to explain the operation of multiple innate immune adaptors, like MAVS, Mydd88 and inflammasomes.^86^ STING SMOCs has more elements than the STING–TBK1–IRF3 model. For instance, experimental data based on CRISPR-Cas9 indicates that (NF-κB essential modifier) NEMO and Inhibitor of nuclear factor kappaB kinase beta (IKKβ) as well as the ubiquitination chain, are required for activation of TBK1 and full NF-κB activation and interferon induction.^79^ This indicates a positive feedback loop between TBK1 and IKKβ to ensure full activation of IRF3 and NF-κB.^79^ In a STING SOMC assumed by us (Fig. 3), kinase TBK1 and IKKβ act jointly and participate in high-dimension signalosome with STING.^87^

Post-transcriptional modifications, such as polyubiquitination, further extend the intensity of the STING SOMCs and recruit more adaptors such as transforming growth factor β-activated kinase 1 (TAK1) and tumor necrosis factor receptor-associated factors (TRAFs). TRAF3 and TRAF6 were also reported to contribute to STING-mediated signaling responses upstream of TBK1. In detail, TRAF6 may be involved predominantly in dsDNA-mediated NF-κB activation rather than IRF3-mediated IFNβ production in mouse embryonic fibroblasts (MEFs), and TRAF3 mainly dedicates dsDNA-mediated noncanonical NF-κB pathway.^79^ The output of this complex may vary due to the differential architecture of SMOCs and can be modulated by artificial intervention.^88^ In more closely related vertebrates, the strength of NF-κB signaling and IFN initiated by STING activation varied drastically among species. For example, zebrafish have evolved a C-terminal extension of STING CTT to strengthen the NF-κB signaling through TRAF6 recruitment.^65^ STING has been also reported to activate MAPKs and STAT6. It is not clear whether MAPK and STAT6 are activated in this framework of STING SMOCs (Fig. 3).

### The autophagy and STING

**Fig. 4:**
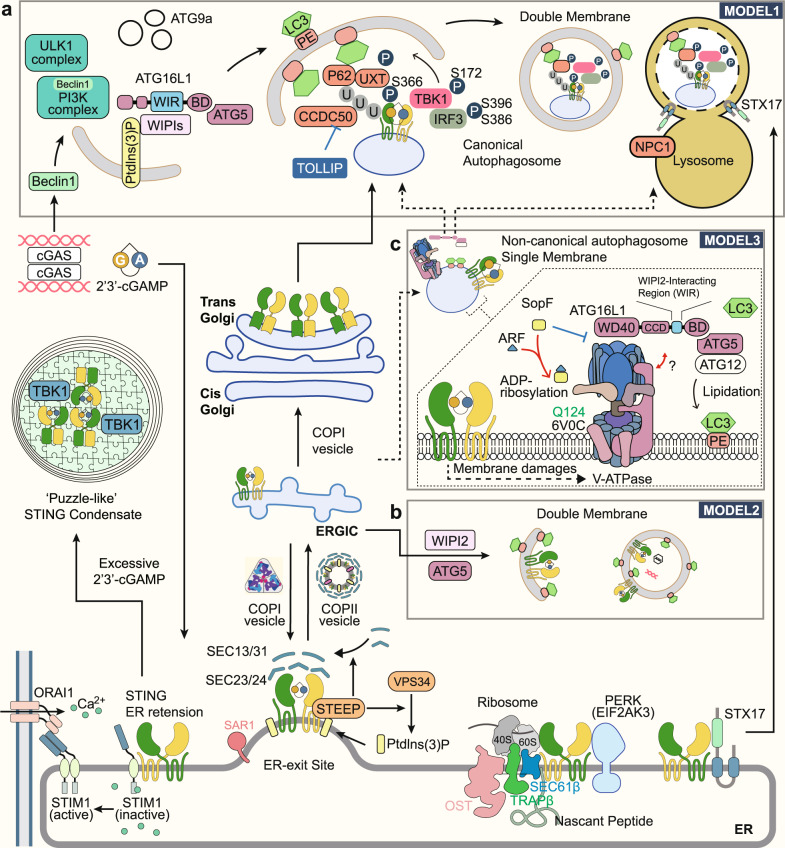
STING trafficking route and its relationship with autophagy. STING activation requires its translocation from ER to the Golgi apparatus, which resembles the early secretory pathway. In a steady state, STING is sequestered on ER membrane by STIM1 and interacts with the translocon complex, PERK, and STX17. STEEP regulated STING exit by promoting COPII assembly and recruiting VPS34 to augment phosphatidylinositol-3-phosphate (PtdIns(3)P) production and ER membrane curvature. There are three models depicting the relationship between STING and autophagy. **a** In model 1, cGAS can induce canonical autophagy, which parallels and negatively regulates STING trafficking. TBK1 can activate the STING–IRF3 axis and induced P65-mediated STING degradation via double-membrane autophagosomes, which eventually fuse with a lysosome. Several autophagy receptors like CCDC50, UXT, and NPC1 mediate STING degradation. **b** In model 2, STING can induce canonical autophagosome formation using ERGIC membrane souce, dependent on both WIPIs and ATG5. This process facilitates the cytosolic clearance of the virus and dsDNA. **c** In model 3, STING activation recruits the V-ATPase–ALG16L1 axis to mediate LC3B lipidation of the single-membrane bacteria-containing vacuole. V-ATPase can sense the damage of the endoplasmic reticulum-Golgi intermediate compartment/Golgi membranes and bind to the ATG16L1 WD40 domain. SopF, a bacterial effector protein, can co-act with ARF1 and inhibit the process by ADP-ribosylating Gln124 of ATP6V0C. ARF: ADP-ribosylation factor; ATG, autophagy-related 1; BECN1, beclin 1; BD, (ATG5) binding domain; CCDC50 coiled-coil domain containing 50; cGAMP cyclic GMP–AMP; cGAS cyclic GMP–AMP synthase; COP coat protein complex, ER endoplasmic reticulum, ERGIC ER–Golgi intermediate compartments, IRF3 interferon regulatory factor 3, LC3 microtubule-associated protein 1 light chain 3, NPC1 NPC intracellular cholesterol transporter 1, Orai1 ORAI calcium release-activated calcium modulator 1, PE phosphatidylethanolamine, PERK PKR-like endoplasmic reticulum kinase, PI3K phosphoinositide 3-kinase, PtdIns(3)P phosphatidylinositol-3-phosphate, STEEP STING ER exit protein, STIM1 stromal interaction molecule 1, STING stimulator of interferon genes, STX17 syntaxin 17, TBK1 TANK-binding kinase 1, TOLLIP Toll-interacting protein, TRAPβ translocon-associated protein subunit beta, ULK1 Unc-51 like autophagy activating kinase 1, UXT ubiquitously expressed prefoldin like chaperone, VPS34 vacuolar protein sorting 34, WIPI WD-repeat protein interacting with phosphoinositides, WIR WIPI2 interacting region

Early in 2009, a close relationship between STING activation and autophagy induction was established for the first time. Activated STING was found to colocalize with autophagy proteins, microtubule-associated protein 1 light chain 3 (LC3), and autophagy-related gene 9a (Atg9a), but not ULK1, Atg5, or Atg14L. But STING-positive vesicles are devoid of morphological characteristics of autophagosomes, double-membrane-bound structures.^89^ Further studies have shown that STING is responsible for autophagy induced by the presence of cytosolic pathogenic DNA,^90^ and this process functions in viral and bacterial clearance,^91^ which is also known as xenophagy. STING-dependent activation of TBK1 has been reported to be responsible for the ubiquitination of bacterial phagosome,^91,92^ and the direct interaction between Beclin 1 and cGAS for the induction of autophagy.^93^ ER stress induced by STING activation was also reported to couple the inactivation of the mechanistic target of rapamycin (mTOR) and ER-phagy. However, the detailed mechanism of DNA sensation to induce autophagy has not reached a consensus.^94^ Around 2018, several different models are proposed (Fig. 4).

In Model 1, intact canonical autophagy, which is initiated by ULK1 dephosphorylation, parallels STING activation along the secretory pathway. These two pathways facilitate the accumulation of double-membrane vesicles and an endosome-like single membrane vesicle, respectively, and converge TBK1 to confer p62 and IRF3 phosphorylation. Phosphorylated p62 can recognize the polyubiquitin chain of STING and recruit it to lysosome-mediated degradation. Thus, canonical autophagy negatively controls STING activation.^95^ In addition, more autophagy receptors were discovered to be involved in this process, such as Coiled-coil domain containing 50 (CCDC50),^96^ NPC intracellular cholesterol transporter 1 (NPC1),^97^ and Ubiquitously expressed prefoldin like chaperone (UXT)^98^ (Fig. 4a).

In Model 2, STING can directly induce non-canonical autophagy independent of the upstream adaptors of canonical autophagy and TBK1.^13^ The study pinpointed that cGAMP induced LC3 lipidation of STING-containing ER-Golgi intermediate compartments (ERGIC) through WIPI2 and ATG5 (Fig. 4b). A small region that spans residues 330–334 of STING, but not the CTT accounting for TBK1 and IRF3 binding, is responsible for autophagy induction.^14^ STING trafficking is essential for the process, which can be affected by siRNA targeting GTPase SAR1A or the component of the coat protein complex II (COPII) SEC24. It was also observed that cGAMP stimulation improved the binding of GTPase ADP-ribosylation factor (ARF) to its effector protein Golgi-localized γ-ear-containing ARF-binding protein 3 (GGA3) and the interaction between STING and SEC24C, which is dependent on L333 and R334.^14^ Targeting ARF by Brefeldin A (BFA), the ARF inhibitor, and Golgicide A, the ARFGEF GBF1 inhibitor, blocked the ER exit of STING.

Meanwhile, a paradigm-shifting breakthrough in xenophagy research introduces a brand new understanding of STING-induced autophagy. STING activation can recruit the V-ATPase–ALG16L1 axis to mediate the lipidation of the single membrane vacuole LC3B, bypassing the requirement of canonical upstream autophagy machinery. The binding of ATG16L1 to V-ATPase is mediated by the WD40 domain of ATG16L1, which is also found in the homologue ATG16L1 of *Nematostella vectensis* (*N. vectensis*), but it is not suitable for canonical autophagy.^88,99^ It is proposed that damage to the ERGIC/Golgi membranes induced by STING or the change in the organelles is sensed by V-ATPase,^100^ which is uncoupled from its H^+^-pumping function. SopF, a *Salmonella* T3SS effector protein, can specifically ADP-ribosylated Gln124 of ATP6V0C in the V-ATPase to block the process. ARF GTPases as a cofactor required for SopF functioning. Although autophagy was induced both in the STING-ΔCTT cells and STING L373A cells, the strength of autophagy appears to be weaker, indicating that TBK1 also plays a boosting but not priming role for STING-related autophagy.^82^ These different forms of autophagy may be jointly involved in STING activation. Furthermore, STING degradation involved a pathway beyond autophagy, as ATG5 deficiency abolished LC3 lipidation but not STING degradation^14^ (Fig. 4c).

### STING and cell death

Cell death induced by gain-of-function STING mutants is first observed in STING-associated autoimmune disease, and apoptosis is shown to be involved.^12^ However, the molecular mechanisms behind STING-related cell death are involved in diverse signal cascades. In a cell-intrinsic way, phosphorylated IRF3 can interact with the pro-apoptotic proteins BAX and BAK and thereby lead to transcription-independent induction of apoptosis.^11^ The paracrine of cytokines after STING activation also effectively render surrounding cell more vulnerable to cell death. It has been reported in certain cells, STING can induce lysosomal cell death through triggering membrane permeabilization of lysosome.^101^ Interestingly, in tumor cells, STING regulates cell death through DNA damage response (DDR) independently of its canonical IFN pathways, wherein STING–TBK1 axis stimulates the autophosphorylation of the DDR kinase ATM, with the consequent activation of the CHK2–p53–p21 pathway and the induction of G1 cell cycle arrest. Ferroptosis is newly identified form of cell death, featured by iron-mediated lipid peroxidation and subsequent plasma membrane ruptures.^102^ STING promotes ferroptosis in human pancreatic cancer cell lines by increasing MFN1/2-dependent mitochondrial fusion, leading to subsequent reactive oxygen species production and lipid peroxidation.^103^ In a reciprocal manner, ferroptotic inducers like high-iron diets or Gpx4 depletion can result in the release of 8-OHG, an oxidized DNA damage product, which is able to activate STING-dependent DNA sensor pathway and drive macrophage infiltration and activation in an oncogenic Kras murine model of spontaneous pancreatic ductal adenocarcinoma (PDAC).^104^

### RNA virus restriction and STING–PERK axis

Although STING is currently recognized as a vital sensor for the DNA virus, substantial data supports it also counts in restricting the RNA virus. As the mechanism is less investigated, this phenotype is less visited by most reviews. It is easy to understand that human immunodeficiency virus (HIV)-1, as a retrovirus, can activate the cGAS–STING pathway through its cDNA generated by the RNA reverse transcription.^105^ However, in the very first papers reporting the identification of STING, data showed that STING effectively controlled RNA virus titers, including Sendai virus, vesicular stomatitis virus (VSV),^1^ and influenza A virus (IAV).^106^ All these viruses belong to enveloped, nonretroviral RNA viruses.

It was reported that enveloped virus-cell fusion can trigger the IRF3-mediated immune response,^107^ which involved the PLC–γ-PI(3)K pathway and the release of Ca^2+^ from the ER.^107–109^ This response can occur when cells are infected with a low-level enveloped virus prior to virus replication, or triggered by nonreplicating virus vectors or synthetic lipid-based carriers,^110^ and even fusion between host cells.^111^ A further study indicated that the signal of membrane fusion activates STING in a cGAS and cGAMP-independent manner. The residues 162–172 in the longest helix are responsible for this signal sensation, among which arginine 168 was indispensable. This arginine mirrors two other arginines R232 and R238 in the STING lid domain, which are essential for 2’3’-cGAMP recognition. The fusion-STING and cGAS–cGAMP–STING pathways can be functionally separated by these mutants.^106^ The 162–172 fragment is also targeted by a viral protein fusion peptide (FP) of the influenza A virus (IAV) to antagonize STING dimerization for immune evasion.^106^ Following this mechanism, it has recently been reported that SARS-CoV-2 spike protein-induced cell fusion activates the cGAS–STING pathway and the interferon response.^112^

Other studies found that STING can also restrict the RNA virus through translation inhibition.^113^ Accordingly, during the first 24 h of infection, cells that are genetically unresponsive to IFN are no more sensitive to VSV infection than their WT counterparts, indicating the existence of other potential defense responses. Inhibition of protein synthesis by STING occurs at the level of translation initiation and restricts the production of viral and host proteins. This pathway of translation inhibition is paralleled to IFN expression, but perhaps in the early stages of an infection, in a cell-intrinsic manner.^113^ A recent study identified the STING–PKR-like endoplasmic reticulum kinase (PERK)–eIF2α pathway, which represses the translation of cap-dependent messenger RNA, but shifts translation to an inflammatory- and survival-preferred program. Mechanically, STING at the ER binds to and directly activates ER-located kinase PERK, which emerged in the early phase before STING ER exit and acted independent of classical STING cascades including TBK1–IRF3 axis, NF-κB, autophagy, and unfolded protein response (UPR). Physiologically, this is also critical to damage-induced cellular senescence and organ fibrosis.^15^

Finally, STING was reported to promote the replication of human rhinoviruses, which are non-enveloped positive-strand RNA viruses, and specially finished their replication in intracellular compartments made of modified host membranes, referred to as replication organelles (ROs).^114,115^

## The spatiotemporal trafficking of STING

The membrane exchange between different organelles is constantly executed for cellular homeostasis and is also tightly regulated. Translocation of STING from the ER to Golgi is the hallmark event of its activation, which resembles the early secretory pathway. Here, we present the details of this process with reference to the STING interactome (Table 1).

**Table 1:** STING interactome

Interacting protein			STING	Types of regulation	Function	Ref.
Protein	No. of TMD	sub-domain for interaction	sub-domain for interaction			
NLRC3	None	Nucleotide-binding domain	LBD (aa139–344)	−	Blocks STING trafficking and STING–TBK1 association	^251^
ZDHHC1	4 TM	N terminus of ZDHHC1 (aa1–271)	TMD	+	Promotes the dimerization and aggregation of STING(R232/H232)	^252^
BTK	None	SH3/ SH2-interaction domain	TMD	+	BTK Deficiency impairs STING-mediated TBK1 and IRF3 activation	^253^
NLRX1	None	Nucleotide-binding domain	N.D.	−	disrupt STING–TBK1 interaction	^254^
iRhom2	7 TM	First TMD	TMD	+	Promotes STING–TRAPβ interaction and stabilize STING through depolyubiquitin (K48)	^255^
S6K1	None	Kinase domain	The phosphorylation site	+	Recruits IRF3 to the STING–TBK1 complex for phosphorylation	^256^
TRIF	None	CTD	CTD	+	Stabilizes the dimeric form of STING	^257^
IFI16	None	PYRIN domain	N.D.	+	Facilitates recruitment of TBK1 to STING and its phosphorylation of STING	^51^
Src	None	SH3 domain	N terminal PXXP motif	+	Promotes the phosphorylation of TBK1 on Tyr179 and TBK1 activation	^258^
TMED2	1 TM	Lumen GOLD and CC domain	TMD	+	Promotes the Recruitment of STING into the COPII Complex and the trafficking	^259^
SNX8	None	N-terminal domain (aa1–180)	TMD	+	Recruits the VPS34 to STING and promote STING trafficking	^260^
UBXN3B	None	UAS domain	N.D.	+	Modulates TRIM56-mediated K63-linked ubiquitination of STING and activation of TBK1	^261^
TMEM203	4 TM	N-terminal TMDs	TMD	+	Promotes STING trafficking and Competes for STING Binding with STIM1	^262^
YIPF5	5 TM	C-terminal TMDs	TMD	+	Facilitates STING recruitment to COPII in the presence of cytoplasmic dsDNA	^263^
STIM1	1 TM	Direct interaction of STIM1	TMD	−	Retains STING at the ER under steady-state conditions	^118^
HER2	None	Intracellular domain	CTD (aa139–379)	−	Recruits AKT1 to phosphorylate TBK1 at S510 and impede STING signalosome assembly	^74^
TOLLIP	None	CTD	Short loop between TMD2 and TMD3	+	Stabilizes STING on the ER under steady-state conditions	^119^
STEEP	None	CTD	TMD and dimerization interphase	+	Facilitates PtdIns(3)P production for ER membrane curvature and COPII assembly	^126^
Notch	1 TM	Notch intracellular domain (NICD)	LBD	−	Inhibits STING activation by competing with cGAMP for the CDN-binding site	^264^
Galectin-9	None	Carbohydrate recognition domain 1	STING	−	Promotes the E3 ubiquitin ligase TRIM29-mediated K48-linked ubiquitination of STING	^265^
DAPK3	None	N.D.	CTD	+	Inhibits STING proteasome-mediated degradation in unstimulated states and promotes STING K63-ubiquitination and STING–TBK1 interaction when activated	^266^
REC8	None	N.D.	The 111–221 section of STING	+	Inhibits the K48-linked ubiquitination (on Lys150 and Lys370) triggered by RNF5	^267^
TMEM120A	6 TM	CTD	N.D.	+	Promotes STING translocation from the ER to ERGIC and its activation	^268^
STX17	2 TM	N.D.	CTD	+	Inhibits autophagosomal fusion with lysosomes by sequestering STX17 at ER/ERGIC	^160^
UNC93B1	12TM	N.D.	N.D.	−	Suppresses STING signaling by targeting STING for lysosome degradation	^269,270^
ALG2	None	N.D.	CTD	−	Inhibits STING trafficking through binding to its C-terminal tail	^271^

*aa* amino acid, *ALG2* apoptosis-linked gene 2, *BTK* bruton tyrosine kinase, *CDN* cyclic dinucleotide, *cGAMP* cyclic GMP–AMP, CTD C-terminal domain, *COPII* coat protein complex II, *DAPK3* death-associated protein kinase 3, *ER* endoplasmic reticulum, *ERGIC* ER–Golgi intermediate compartments, *HER2* human epidermal growth factor receptor 2, *IFI16* interferon gamma inducible protein 16, *IRF3* interferon regulatory factor 3, *iRhom2* inactive rhomboid 2, *LBD* ligand binding domain, *N.D.* not determined, *NLRC3* NLR family caspase recruitment domain containing 3, *NLRX1* NLR family member X1, *NTD* N-terminal domain, *REC8* REC8 meiotic recombination protein, *RNF5* ring finger protein 5, *S6K1* p70 ribosomal protein S6 kinase 1, *SNX8* sorting nexin 8, *STEEP* STING ER exit protein, *STIM1* stromal interaction molecule 1, STX17 syntaxin 17, *TBK1* TANK-binding kinase 1, *TMED2* transmembrane p24 trafficking protein 2, *TMEM* transmembrane protein, *TOLLIP* Toll-interacting protein, *TRAPβ* translocon-associated protein subunit beta; TRIM29, tripartite motif containing-containing protein 29, *TRIF* TIR domain containing adaptor protein inducing interferon-beta, *UBXN3B* UBX domain-containing protein 8, *UNC93B1* Unc-93 homolog B1, *YIPF5* Yip1 domain family member 5, *ZDHHC1* zinc finger DHHC-type containing 1, *PtdIns (3)P* phosphatidylinositol-3-phosphate

### Steady retention of STING in ER

In 2009, Barber first identified components of the ER translocon complex, TRAPβ and SEC61β, that interact with STING and maintain its normal function. The translocon mainly conducts the translocation of nascent peptides into the ER lumen or their integration into the lipid membrane and co-translationally facilitates additional processes for protein maturation.^116^ The function of these interactions is unclear.

How is STING prevented from being captured into a vesicle in a steady state is not clear. In the current model, the ligation of 2’3’-cGAMP causes STING ER exit, which may be involved a coordinately use of a hierarchy of adapters, receptors, and accessory factors. As the classification sequences for ER retravel, such as the KDEL and dilysine motifs, were not discovered in the STING structure,^117^ the substantial conformational change in the activation of STING may release some potential signal for the ER exit, while some mutants are believed to have a lower threshold in this process. Thus, retention signals and extensive interactions among resident chaperones of the ER could prevent some proteins from entering vesicles.

The resident protein of the ER stromal interaction molecule 1 (STIM1) was reported to interact directly with STING to mediate its retention in the ER.^118^ Deficiency in STIM1 strongly enhanced the expression of type I IFNs in a STING-dependent way, which accounts for autoimmune complications in patients with the STIM1 mutation. Their interaction is mutually maintained, as the biochemical association between them was reduced by stimulation of STIM1 or STING. The expression level of STING also, in turn, regulates the function of STIM1 in cellular Ca^2+^ modulation, although with cell-type specificity.^118^ TOLLIP is another stabilizer of STING through direct interaction to prevent degradation mediated by lysosomes. Mechanically, TOLLIP deficiency results in STING degradation by hyperactivating the IRE1 ER stress sensor IRE1α. TOLLIP was originally reported to mediate the clearance of Huntington’s disease-linked polyQ protein aggregates. PolyQ proteins in the Huntington’s disease mouse striatum can sequester TOLLIP away from STING, leading to reduced STING protein and dampened immune signaling.^119^

### The translocation of STING between ER/Golgi and to lysosome

COPA syndrome is a recently discovered autoimmune disease with prominent type I interferonopathy, caused by a monogenic mutation in the N-terminal WD40 domain of COP-α (a component of coat protein complex I, COPI). Studies reported a model in which impaired COPI transport induced the activation of STING through forced Golgi localization of STING.^120–123^ Surf4 was confirmed to bridge the recognition of COP-α towards STING.^122^ However, mammalian cells have additional recycling stations between ER and Golgi, coined ERGIC.^124^ The COPI formed in ERGIC can send ER-resident proteins, which contain retrieval signal, back to ER or further differentiate the ERGIC into pre-Golgi intermediates (Fig. 4), making the role of COPI more ambiguous. A detailed mechanism of the COPA syndrome needs further investigation.

COPII-coated vesicles comprise five subunits: Sar1-GTP, dimeric Sec23/Sec24 inner coat, and tetrameric Sec13/Sec31 outer cage. The assembly of COPII coat proteins occurs in membrane regions known as ER exit sites. The GTPase Sar1 recruits Sec23/24 to the ER for selection of cargo proteins, to which Sec13/31 is recruited through direct interactions and drives membrane bending. STING as the integral membrane cargo protein is, in principle, accessible to coat adaptors. Sec24 selects cargo proteins by binding directly to ER export signals.^125^ The mutation assay demonstrated that the residues L333 and R334 in STING are responsible for the interaction of STING with SEC24C at early time points.^14^ The motif between aa343 and aa354 was also reported to be involved in the initiation of STING ER exit, even for the GOF mutant V155M.^83^

STEEP is a novel protein recently discovered in the STING interactome that contributes to STING ER exit. On the one hand, STEEP enables the embedment of SAR1 into ER, thus facilitating COPII assembly. On the other hand, STEEP recruits VPS34 to the ER to increase phosphatidylinositol-3-phosphate (PtdIns(3)P) production and ER membrane curvature formation.^126^ STEEP’s function to promote the exit of STING from the ER is required for the STING-induced expression of IFN and IL-6 and the activation of autophagy. As for VPS34, its requirement for STING-induced IFN production was reported productively by siRNA-mediated VPS34 knockdown.^127^

It was proposed that STING interacts with TBK1 in Sec5-containing endosome compartments.^4^ RNA interference of Sec5 significantly impaired IFN induction.^4^ The Shigella effector protein IpaJ and BFA potently inhibit ARF1 GTPase and greatly dampen STING-induced IFN production, while VirA that disrupts post-ERGIC vesicle transport does not present an impact. Based on these data, STING signal transduction already takes place at the ERGIC. Actually, such a common supposition that BFA blocks export from the ER is a long-standing overinterpretation. Consistent with the absence of ARF1 from the ER, there is no evidence that BFA directly interferes with the assembly of COPII coats.^124^ In line with this, the interaction between Sec24 and STING was not inhibited by BFA.^126^ Caution should be exercised when interpreting the subcellular compartment where BFA arrests STING transport.

A recent study using whole-cell FIB-SEM technology pinpoints that, rather than vesicles alone, the ER spawns an elaborate, interwoven tubular network of contiguous lipid bilayers for protein export.^128^ COPII remains on ER exit sites (ERESs) to select and concentrate exported cargo rather than coating Golgi-bound carriers,^129,130^ while COPI and other ARF1 effectors may instead directly control cargos exit from ERESs.^130^ It is not clear whether STING is translocated through this structure.

In the later phase of STING activation, STING will be translocated into the lysosome for degradation, which serves as a negative feedback mechanism to ensure cascade termination and avoid continued activation. NPC1 was reported as a lysosomal adaptor for STING that mediates the recognition and degradation of STING.^97^ However, in certain cells such as HEK293T or BLaER1 monocyte cells, activated STING traffics to the lysosome, where it is not degraded but triggers membrane permeabilization. The lysis of the lysosomes and the leakage of the lysosomal content into the cytosol thus lead to lysosomal cell death (LCD) and NLRP3 activation.^131^

### The recruitment of TBK1 to STING

Coimmunoprecipitation and immunostaining in intact cells suggest that a considerable amount of TBK1 forms a constitutive interaction with STING in the absence of cGAMP, and this interaction can be further enforced by cGAMP stimulation. However, in the in vitro pull-down assay, this interaction was more prominent and did not show improvement after adding cGAMP, indicating the incomplete accessibility of TBK1 to STING in intact cells. In both conditions, the interaction can be abolished by mutation of key residues in TBM or counterpart residues in TBK1 dimers for STING CTT binding. Thus, STING CTT may be sequestered in steady state in unknown mechanism and be released when activated.^40^ Detailed explanation on how TBK1 is recruited into STING still requires further investigation.

### The phase separation of STING

Liquid–liquid phase separation or phase condensation of biomacromolecules is an important biological phenomenon that has received great attention recently. It helps to organize complex biochemical reactions in a relatively dense space. Such higher-order assemblies have recently emerged as an important mechanism for facilitating signal transduction. A recent study has reported that STING can also undergo phase separation in the endoplasmic reticulum, which is induced by excessive 2′3′-cGAMP and prevents innate immunity from overactivation.^17^ Under electron-microscopy, it presents to be micrometer-sized ‘puzzle’-shaped condensates with highly organized membranous structures in the ER, which differ from the morphology of activated STING, the submicrometre-sized puncta in the perinuclear compartment. Treatment with BFA and CTT deletion also did not inhibit condensation. The residue 309–342 is the intrinsically disordered region (IDR) required for the condensation of STING, where two conserved residues, E336 and E337, appeared to be the most important. TBK1 is recruited to STING condensate, whereas IRF3 is insulated from it (Fig. 4). Intriguingly, the TBK1 captured in STING condensates is not phosphorylated. It is proposed that two routes are there to form the STING condensate. One emerged from a highly organized annulate lamella that release membranes to the inner zone and built up the puzzle-like structure gradually. In the other route, very compacted ER granules transformed into puzzle-like structures. It is unclear what biochemical process dictate the STING condensation. But the annulate lamella in STING condensate is similar to another simultaneously reported structure of ER, which is termed ER whorls. This structure, induced by ER stress, contains ER-resident proteins such as the Sec61 complex and PERK, and is mediated by PERK kinase activity and COPII machinery.^132^ It should be noted that these mediators in ER whorls formation are also closely related to STING, easily conjuring up the potential relationship between them. In the future, more rigorous studies are needed to investigate the detailed mechanism and physiological function of STING condensation.

Although IRF3 is left out of the STING condensate, it can also form cellular condensates in another scenario. Neurofibromin 2 (NF2) is a tumor suppressor, but can result in frequent tumorigenesis when missense mutation occurs. The mutated NF2 gains extreme associations with IRF3 and TBK1 to form cellular condensates. Similarly, this condensate also suppresses STING signaling through eliminating TBK1 phosphorylation and abolishes antitumor immunity initiated by STING in mice.^133^

## The regulation of STING expression

### Genetic control of STING pathway

The STING gene is located on chromosome 8 and is found to be ubiquitously expressed in a variety of cells, except in cells such as neutrophils and NK. STING signaling is commonly suppressed in a wide variety of cancers, predominantly through epigenetic silencing of promoter regions and loss-of-function mutation.^134^

In KRAS-driven lung cancer, the LKB1 mutation represses STING expression by increasing DNMT1 and EZH2 activity, which target the methylation and modification of the H3K27Me3 of the STING promoter, respectively.^135^ In triple-negative breast cancer, MYC could activate DNMT1 transcription and induce DNA methylation within the 5’-untranslated region of STING to suppress STNG expression.^136^ LncRNA nuclear paraspeckle assembly transcript 1 (NEAT1) can also bind to DNMT1 and suppress STING for tumor evasion.^137^

The demethylating agent 5-aza-2’-deoxycytidine (5AZADC) can recapitulate STING expression,^138^ which would also promote MHC-I-mediated tumor antigen presentation and T cell recognition in tumor cells.^139^ IFN-α has been reported to increase STING expression through a STAT1 binding site on the STING promoter.^140,141^ In type 2 immune environment, STING expression in epithelial cells of nasal tissue was negatively regulated by IL-4 and IL-13 in a STAT6-dependent manner.^142^

At the mRNA level, miR-181a directly targeted the conserved binding site in the 3’-UTR of STING mRNA and decreased the level of STING mRNA.^143^ The demethylated form of hnRNPA2B1A, a newly identified nuclear innate sensor, can promote nucleo-cytoplasmic trafficking of cGAS, IFI16, and STING messenger RNAs for expression priming. Additionally, N6-methyladenosine (m6A) in mammalian mRNAs can promote mRNA translocation from the nucleus to the cytoplasm. Concordantly, IFN expression was impaired by METTL3 knockout, with lower levels of m6A of cGAS, p204 and Sting^144^ as well as Irf3.^145^ Reciprocally, METTL3 activity can be enhanced by its phosphorylation of S67 mediated by the STING–TBK1 axis, which underlying a positive feedback circuit.^145^ As an m6A eraser, fat mass and obesity-associated (FTO) knockdown leads to increased IFN expression in HSV-1 infection.^144^

### Alternative splicing

Up to now, there are six alternative splicing isoforms of STING, reviewed elsewhere.^146^ Interestingly, an isoform that lacks the transmembrane domain in its N-terminus was recently reported to locate on the plasma membrane, directly sensing the extracellular cGAMP and inducing IFN,^147^ incidentally mirroring the first report of STING on the cell membrane in 2008.^10^ However, how is the signal relayed to the nucleus remains enigmatic. The RNA-binding protein LUC7L2 down-regulates the level of the STING protein by directly binding to its precursor messenger RNA and inhibiting its splicing.^148^

### Post-translational modifications of STING

In addition to the genetic modulation of STING transcription, the post-translational modification is much weighted in the modulation of STING. It has been studied extensively and is reviewed elsewhere.^149^ However, new clues were added to this area in recent years.

**Fig. 5:**
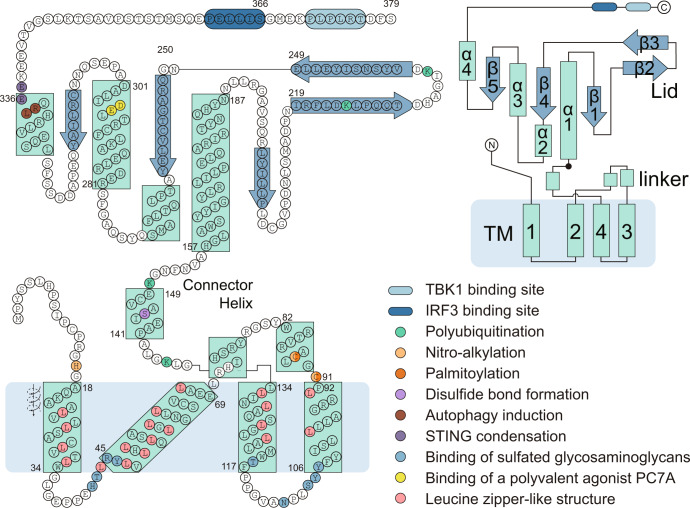
Domain composition and sequence of human STING. Functional residues are marked with colors and annotated in the right-bottom panel. The arrangement of α-helix and β-strands is annotated in the right-upper panel. PC7A polymer with a cyclic seven-membered ring^62^

Here, we vividly illustrate the residues that are subjected to modifications in the STING structure model (Fig. 5) and summarize the function of these modifications (Table 2). The modification types mainly include polyubiquitination and phosphorylation, and to a lesser extent, sumoylation, palmitoylation, nitro-alkylation, oxidation, carbonylation, and disulfide bond formation. Polyubiquitination of different types will dictate the contrasting effect on STING. It was accepted that K48-polyubiquitination mainly facilitates proteosome-mediated degradation.^150,151^ On the contrary, K63 polyubiquitination usually promotes STING complex formation.^152,153^ The YAP/TAZ hippo signaling components associate directly with and repress TBK1 by preventing Lys63-linked ubiquitylation of TBK1.^154^ Hippo pathway activation leads to phosphorylation and degradation of YAP/TAZ through Lats1/2 kinases, thus alleviating inhibition against the STING cascade.^154^

**Table 2:** STING post-translational modifications

Type of PTMs	Residues	Enzyme	Types of regulation	Functions	Ref.
Polyubiquitination(K6)	K20	TRIM13	+	Promotes STING degradation through ERAD pathway.	^272^
Polyubiquitination(K11)	K150	RNF26	+	Stabilizes STING through K11-linked polyubiquitination	^273^
Polyubiquitination(K27)	K137/K150/K224/K236	AMFR	+	Promotes recruitment of TBK1	^274^
Polyubiquitination(K48)	K150	RNF5	−	Promotes degradation of STING in a proteasome pathway	^275^
Polyubiquitination(K48)	K275	TRIM30α	−	Promotes K48-linked ubiquitination of STING and its degradation	^150^
Polyubiquitination(K48)	K370	TRIM29	−	Promotes K48-linked ubiquitination of STING and its degradation	^151^
Polyubiquitination(K48)	K288/K337	TRIM29	−	Promotes proteasome-dependent degradation of STING	^276^
Polyubiquitination(K63)	K150	TRIM56	+	Promotes dimerization of STING and recruitment of TBK1	^152^
Polyubiquitination(K63)	K20/K150/K224/K236	TRIM32	+	Promotes interaction with TBK1	^153^
Polyubiquitination(K63)	K224/K236/K289/K338	MUL1	+	Promotes dimerization and trafficking of STING	^277^
Polyubiquitination(K63)	K20/224/289	RNF115	+	Promotes the oligmerization of STING and the recruitment of TBK1	^278^
Polyubiquitination(K63)	N.D.	LMO7	+	Promotes K63-linked STING poly-ubiquitination and STING–TBK1 interaction	^266^
Polyubiquitination(K63)	N.D.	TRIP12	+	Promotes K63-linked STING poly-ubiquitination and STING–TBK1 interaction	^266^
Deubiquitylation (K63)	K150	MYSM1	−	Interacts with STING to cleave STING ubiquitination and attenuate the pathway	^279^
Deubiquitylation (K27)	N.D.	USP13	−	Prevents recruitment of TBK1	^280^
Deubiquitylation (K27)	N.D.	USP21	−	Inhibits the formation of STING-TBK1-IRF3 complex	^281^
Deubiquitylation (K48)	N.D.	USP20	+	Deploited by USP18 to Stabilize STING	^282^
Deubiquitylation (K48)	N.D.	CYLD	+	Stabilizes STING by removing the K48-linked polyubiquitin chains	^283^
Deubiquitylation (K48)	N.D.	EIF3S5	+	Stabilizes STING by removing the K48-linked polyubiquitin chains	^255^
Deubiquitylation (K48)	K347	OTUD5	+	Stabilizes STING stability	^284^
Deubiquitylation (K48)	N.D.	USP35	+	Removes K6-, K11-, K27-, K29- or K63-linked polyubiquitin chains from STING	^285^
Deubiquitylation (K63)	N.D.	USP21	−	Inhibits the formation of STING–TBK1–RF3 complex	^281^
Phosphorylation	Y245	Src	+	Enhances the activation of STING	^286^
Phosphorylation	Y245	EGFR	+	Promotes STING relocation to late endosome for IRF3 activation and stabilizes STING	^287^
Phosphorylation	S358	TBK1	+	Facilitates recruitment and activation of IRF3	^7,288^
Phosphorylation	S366	TBK1	+	Provides docking site for IRF3	^7^
Phosphorylation	S366	ULK1	−	Facilitates degradation of STING	^127^
Dephosphorylation	Y245	PTPN1/2	−	Promotes degradation of STING in a proteasome pathway	^289^
Dephosphorylation	S358	PPM1A	−	Dephosphorylates both STING and TBK1 and Impairs STING aggregation	^290^
Dephosphorylation	N.D.	PPM1G	−	Dephosphorylates STING but not TBK1	^291^
SUMOylation	K338	TRIM38	+	Promotes oligomerization and recruitment of IRF3 and stabilizes STING	^292^
De-SUMOylation	K338	SENP2	−	Facilitates degradation of STING	^292^
Palmitoylation	C88/91	ZDHHC3/7/15	+	Promotes polymerization and type I Interferon production	^293^
Nitro-alkylation	C88/C91/H16	N.D.	+	Antagonizes palmitoylation and impairs STING signaling	^179^
Disulfide bond	C148	−	+	Promotes polymerization and activation of STING	^177^
Oxidation	C148	−	−	Prevents polymerization and activation of STING	^175^
Carbonylation	C88	GPX4	−	Inhibits its trafficking from the endoplasmic reticulum to the Golgi complex	^180^

*AMFR* autocrine motility factor receptor, also known as gp75, RNF45, *CYLD* CYLD Lysine 63 deubiquitinase, *ZDHHC3/7/15* zinc finger DHHC domain-containing protein 3/7/15, *EGFR* epidermal growth factor receptor, *EIF3S5* eukaryotic translation initiation factor 3 subunit F, *ERAD* ER-associated protein degradation, *IRF3* interferon regulatory factor 3, *GPX4* glutathione peroxidase 4, *LOM7* LIM domain only protein 7, *MUL1* mitochondrial E3 ubiquitin protein ligase 1, *MYSM1* Myb like, SWIRM and MPN domains 1, *N.D.* not determined, *OTUD5* OTU deubiquitinase 5, *PPM1A* protein phosphatase magnesium-dependent 1 delta, *PPM1G* protein phosphatase magnesium-dependent 1 gamma, *PTPN1/2* tyrosine-protein phosphatase non-receptor type 1/2, *RNF* ring finger protein, *SENP2* sentrin-specific protease 2, *STING* stimulator of interferon genes, *TBK1* TANK-binding kinase 1, *TRIM* tripartite motif containing-containing protein, *TRIP12* thyroid hormone receptor interactor 12, *ULK1* Unc-51 like autophagy activating kinase 1, *USP* ubiquitin-specific-processing protease.

A study systematically investigated the deubiquitinating enzymes (DUBs) family in regulating antiviral immunity, and demonstrated six different modes of action of DUBs in type I IFN regulation, two of which involve novel mechanisms.^155^ Similarly, another paper mapped more than 450 protein-protein interactions for 21 endoplasmic reticulum (ER)-bound E3 ligases, which identified that RNF26 co-assembles with TMEM43, ENDOD1, TMEM33, and TMED1 to form a new modulatory axis of STING signaling.^156^

The cleavage of STING is currently reported to be mediated by some pathogen virulence factors, such as proteases encoded by ZIKV, dengue virus, West Nile virus, and Japanese encephalitis virus.^157^ The cleavage of STING by endogenous protein has not been reported.

## STING and cellular metabolism

**Fig. 6:**
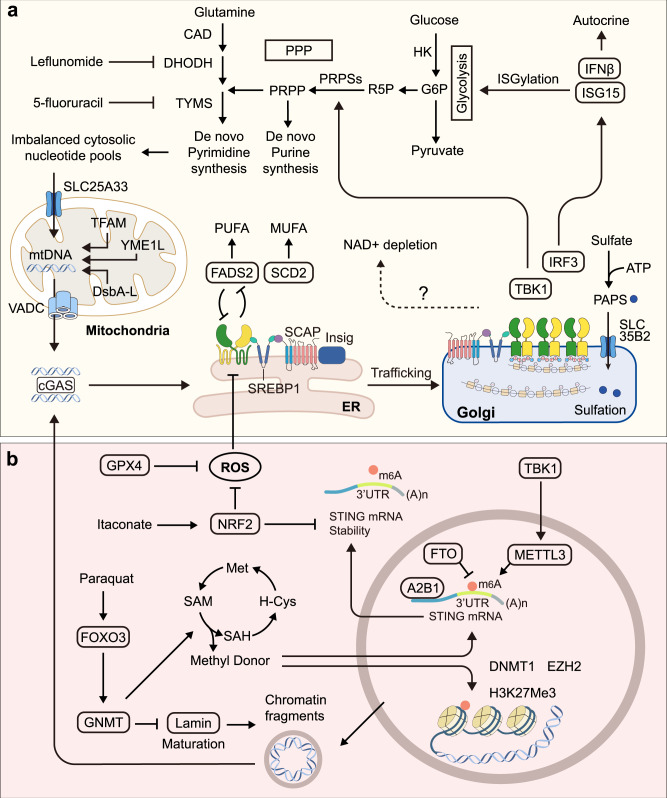
The relationship between STING and metabolism. **a** Nucleic acid and lipid metabolism and STING. Imbalanced cytosolic nucleotide pools can trigger the mitochondrial stress and mtDNA release via VDAC, which activates the cGAS–STING pathway. Interestingly, STING-mediated TBK1 activation can also promote PRPP synthesis by activating the PRPSs. The activated IRF3 dimer enters the nucleus and induces transcription of interferon and ISGs like ISG15. IFN can act in an autocrine way and induce metabolic reprogramming. ISG15 is reported to inhibit glycolysis by covalently modifying the multiple key enzymes. STING activation in tumor cells can induce a decline of NAD^+^ with the known mechanism. **b** Redox balance and STING. Direct delivery of oxidants such as hydrogen peroxide, hypochlorous acid, diamide, and respiratory chain-based ROS inducers such as rotenone, menadione can induce overwhelming ROS can dampen the STING activation. The intracellular antioxidant system GPX4 can maintain the redox balance and normal STING activation. In addition, NRF2 negatively regulates STING expression by decreasing STING mRNA stability. Oxidative stress activates FOXO3 and its transcriptional target GNMT. Reduced intracellular SAM availability induced cytosolic release of chromatin fragments and subsequent cGAS-STING activation via disrupting carboxymethylation and maturation of nuclear lamin. A2B1: hnRNPA2B1A; CAD carbamoyl-phosphate synthetase 2, aspartate transcarbamylase, and dihydroorotase, DHODH dihydroorotate dehydrogenase, DNMT1 DNA methyltransferase 1, DsbA-L disulfide bond A oxidoreductase-like protein, DUBs deubiquitinating enzymes, ER endoplasmic reticulum, FTO fat mass and obesity-associated gene, GPX4 glutathione peroxidase 4, G6P Glucose 6-phosphate, GNMT glycine-N-methyltransferase, HK hexokinase, IFNβ interferon beta, IRF3 interferon regulatory factor 3, ISG interferon-induced genes, Met methionine, METTL3 methyltransferase-like 3, m6A N6-methyladenosine, MUFAs monounsaturated fatty acids, H-Cys homocysteine, H3K27me3 trimethylation of lysine-27 in histone 3, NAD+ nicotinamide adenine dinucleotide, NRF2 nuclear factor erythroid 2-related factor 2, PPP pentose phosphate pathway, PRPP phosphoribosyl pyrophosphate, PRPSs phosphoribosyl pyrophosphate synthetases, ROS reactive oxygen species, R5P ribose 5-phosphate, SAH S-adenosylhomocysteine, SAM S-adenosylmethionine, SREBP2 sterol-regulatory element binding protein 2, SCAP SREBP-cleavage activating protein, STING stimulator of interferon genes, TBK1 TANK-binding kinase 1, TFAM mitochondrial mtDNA-binding protein transcription actor A, TYMS thymidylate synthetase, PAPS 3’-phosphoadenosine-5’-phosphosulfate, PUFAs polyunsaturated fatty acids, VDAC voltage-dependent anion channel

STING activation has been reported to participate in many metabolic diseases, such as obesity and atherosclerosis. However, it is frequently ascribed to its cytokine-inducing ability. Inflammation plays a vital role at the systemic metabolism level. Cytokines can also rewire cellular metabolism through their cognate receptors. For example, IFN acts through IFNR to reprogram cholesterol homeostasis.^158^ Activation of TLR signaling leads to a decrease in cholesterol efflux, which results in further cholesterol accumulation and amplification of inflammatory responses.^159^ The execution of molecular events of the innate immune is highly dependent on the supply of energy. Meanwhile, the re-wiring of the cellular metabolic condition can also shunt the innate immune response to some extent (Fig. 6).

However, recent research indicates that STING can also directly impact cellular metabolism, which is still in the moonlight of the cytokine-mediated effect. Furthermore, in highly differentiated cells, such as islet cells, and skeleton muscle cells, STING can modulate insulin secretion and glucose consumption.^160,161^

### Nucleic acid metabolism and STING

In homeostasis, the cytoplasm is devoid of free DNA due to the presence of multiple enzymes, including TREX1, SAMHD1, IFIH1, ADAR1, RnaseH2, and the endonuclease complex.^162^ Their defects lead to aberrant cGAS-mediated activation of STING. Moreover, exposure to mtDNA induced by mitochondrial stress is another cell-intrinsic trigger for the activation of cGAS–STING. Several mitochondria-resident proteins maintain mitochondrial integrity. The mitochondrial mtDNA-binding protein transcription actor A (TFAM) regulates nucleoid architecture, abundance, and segregation. TFAM deficiency can promote the escape of mtDNA into the cytosol.^163^ In addition, knockout of the disulfide bond A oxidoreductase-like protein (DsbA-L), a chaperone-like protein in the mitochondrial matrix, altered mitochondrial function and promoted mtDNA release.^164^ In non-apoptotic cells, mtDNA is released mainly via pores formed by the voltage-dependent anion channel (VDAC) oligomers in the mitochondrial outer membrane.^165^

mtDNA-mediated STING activation is also under metabolic control. The mitochondrial protease YME1L preserves pyrimidine pools by supporting de novo nucleotide synthesis and proteolysis of the pyrimidine nucleotide carrier SLC25A33. Stabilization of SLC25A33 and inhibition of de novo pyrimidine synthesis induced YME1L deficiency, which is sufficient to separately trigger mtDNA-dependent immune responses.^166^ In WT cells, thymidylate synthase inhibitor 5-fluoruracil can induce robust expression of ISG, while the dihydroorotate dehydrogenase inhibitor leflunomide triggers a mild response.^166^ Genetic down-regulation of the multifunctional biosynthetic enzyme CAD to decrease pyrimidine levels in cells also broadly induced ISG expression.^166^ Phosphoribosyl pyrophosphate (PRPP) derived from the pentose phosphate pathway (PPP) is the vital building block for nucleotide synthesis. Interestingly, STING-mediated activation of TBK1 can also promote PRPP synthesis by activating phosphoribosyl pyrophosphate synthetases (PRPSs) through phosphorylation in T228^167^ (Fig. 6a).

### Reciprocal relationship between lipid metabolism and STING

Accumulating evidence suggests innate immunity has an intimate and reciprocal relationship with cellular metabolism.^168,169^ Several adaptor proteins are confirmed to directly interact with components of cellular metabolism, greatly extending their functions beyond controlling immune responses through the production of cytokines and chemokines. For instance, it has been reported that the RIG-I–MAVS pathway as the major RNA sensor can downregulate glycolysis by disrupting the mitochondria localization of hexokinase 2.^170^ On the contrary, lactate as the product of anaerobic glycolysis can directly bind to MAVS transmembrane (TM) domain and prevent MAVS aggregation.^170^ Furthermore, Myd88, the adaptor of Toll-like receptors, can upregulate glycolysis via TBK1.^88^ Such cases well support the view that immunity and metabolic homeostasis are tightly interconnected, further provoking whether STING is also closely related to cellular metabolism. Interestingly, a rapidly growing body of evidence demonstrates a key relationship between STING and lipid metabolism.

Sterol regulatory element binding protein 2 (SREBP2) is the master transcriptional regulator of cholesterol biosynthesis, which forms a complex with the SREBP-cleavage activating protein (SCAP) in steady state. When SCAP senses ER cholesterol depletion, the SCAP–SREBP2 complex translocates from the ER to the Golgi apparatus for proteolytic activation, quite resembling the process of STING activation. STING can interact directly with SCAP or SREBP2 through the transmembrane domain. In steady state, the knockout of SCAP/SREBP or the silencing of mevalonate kinase (MVK) and HMG-CoA reductase (HMGCR) can elicit a spontaneous induction of IFN in a cGAS-STING-dependent way, though at a relatively low level.^97,158^ It is postulated that perturbations in the pool size of synthesized cholesterol may expose the cGAS ligand, as the replenishment of cholesterol attenuates the IFN signature in SREBP2-null cells.^158^

However, in the STING-activated environment, SCAP as an adaptor facilitates the assembly of STING and the recruitment of TBK1, and SCAP or SREBP2 knockout could impair the full production of IFN.^171^ Specifically, in NPC1 knockout cells, IFN production was significantly enhanced via a spontaneous translocation of SREBP2–SCAP due to the imbalanced cholesterol distribution.^97^ Mechanically, SREBP2 primes STING signaling by ‘tethering’ the STING trafficking from the ER to the Golgi. In this case, the SREBP2 knockout attenuates IFN hypertension^97^ (Fig. 6a).

The forementioned phenotype can also be recapitulated in vivo. Conditional knockout of SCAP in macrophage renders the mice resistant to intranasal infection of murine gamma herpesvirus 68 (MHV68),^158^ while in shRNA-mediated SCAP knockdown mice, the innate immune response against intravenous infection with HSV-1 was severely impaired.^171^ The discrepancy may arise from the different knockout strategies and types of infection, as systemic knockout of SCAP may elicit a profound effect beyond STING.

STING can also interact directly with FADS2 and inhibits FADS2-dependent desaturation of polyunsaturated fatty acids (PUFAs).^16^ STING ablation and agonist-mediated degradation can increase FADS2-associated desaturase activity and leads to the accumulation of PUFA derivatives that drive thermogenesis. This mechanism may explain why the metabolic improvement in STING knockout mice, presented as increased food intake, decreased liver gluconeogenesis, increased energy expenditure and oxygen consumption, and improved insulin-independent glucose management, but not change in circadian rhythm and spontaneous locomotor activity.^16^

In the CD4^+^ T cell compartment, inhibition of the fatty acid synthesis pathway through ACC2, SCD2, or FADS2 triggers the spontaneous production of type I IFN.^172^ An altered cellular lipid profile resulting from VPS13C depletion causes elevated mitochondrial DNA in the cytosol and impaired STING degradation in lysosomal.^173^ The relationship between STING and lipid metabolism confer more specified function in highly differentiated cells warrants further investigation.

### Redox balance and STING

STING activation is also regulated by cellular redox level, which involved the production of reactive oxygen species (ROS) and the perambulation of the intracellular antioxidant system such as glutathione peroxidase 4 (GPX4), nuclear factor erythroid 2-related factor 2 (NRF2).^174^ The thiol in cysteine, as the nucleophilic group, can be attacked by a range of electrophilic reagents. Several functional cysteines in STING like C64, C88, C91, C148, and C206 are susceptible.

Overwhelming ROS can dampen STING activation. Different residues are reported to be subjected to ROS oxidization. In the milieu of menadione treatment, ROS can directly oxidate C147 in mSTING (equivalent to C148 in human STING) to inhibit its polymerization.^175^ On the contrary, another team established that C148 oxidation is constitutive, whereas C206 oxidation is inducible for STING repression.^176^ This study casts doubt on a previous proposal that C148 is involved in a ligand-inducible disulfide bond that stabilizes polymeric STING.^177^ Instead, it stressed the important role of C206 in modulating STING activity. C206 could be in an interaction with a protein partner yet to be identified. Of note, almost all oxidants induced the formation of a non-functional STING polymer,^175,178^ which is different from the active STING polymer. NO2-FAs, formed by iNOS in viral infection, can attack C88, C91, and H16 of STING, then blocking STING palmitoylation and downstream signaling.^179^

Lipid peroxidation caused by GPX4 deficiency can curtail STING activity by producing 4-hydroxynonenal (4-HNE). This end product of lipid peroxidation targets the C88 and C257 of STING for carbonylation, which specifically blocks the Golgi translocation of STING^180^ (Fig. 6b). In addition, RSL3, an inhibitor of GPX4, also targets SELK to suppress IFN production in a lipid peroxidation independent manner, whereas the detailed mechanism is unclear.^180^

The impact of oxidative stress on STING signaling is also cell-type specific and facilitates STING activation in some contexts. For instance, dendritic cells (DCs)-derived ROS triggers SENP3 accumulation, which in turn promotes IFI204-mediated cytosolic DNA detection in a cGAS-independent manner. This mode of STING activation increases DCs antitumor function.^181^ Probably, H_2_O_2_ treatment did not repress STING activation in fibroblasts but rather had a slightly boosting role.^175^

## STING-related diseases

### STING in cancer

As the immunological enhancement of STING, it represents a highly attractive and promising target for cancer immunotherapy (Table 3). Direct activation of STING in the tumor microenvironment leads to potent and systemic tumor regression and immunity.^182^ Myriad immune cell types including dendritic cells, macrophages, natural killer (NK) cells, and CD4 and CD8 T cells are responsive to the antitumor effect induced by STING. Tumor-derived DNA can be engulfed by tumor-infiltrating DCs, promoting tumor-specific antigen presentation and cytotoxic T cell activation in a STING-dependent way.^183^ Furthermore, activation of the STING signaling cell intrinsically improves differentiation and antitumor functions of Th1 and Th9 cells by increasing their respective production of interferon-gamma (IFN-γ) and interleukin-9.^184^ STING has been reported to maintain CD8^+^ T cell stemness by upregulating TCF1 expression and halting Akt activity.^185^ In response to STING agonists, NK cells also mediate the clearance of CD8+ T cell-resistant tumors. As such, the activation of STING could convert the tumor microenvironment to be immunologically active and recruit more immune cells.

**Table 3:** STING-associated diseases

Type of disease or condition	Specific disease	Mechanism of disease related to STING	Refs.
Tumor	Gliomas	STING is epigenetically silenced in gliomas in a developmentally conserved way and can be rescued by methyltransferase inhibition	^294^
	Tumors with defective mismatch repair (dMMR)	Loss of the MutLα subunit MLH1 generates the release of nuclear DNA into the cytoplasm, activating the cGAS–STING pathway	^295,296^
	Triple-negative breast cancer	STING–TBK1–IRF3 pathway activation in cancer cells governs CD8+ T cell recruitment and antitumor efficacy	^297^
	Merkel cell carcinoma (MCC)	STING deficiency contributes to the immune suppressive nature of MCC	^104^
	Pancreatic tumorigenesis	Ferroptotic damage promotes pancreatic tumorigenesis through a STING-dependent pathway	^298^
Viral Infection	HBV infection	The physiological lack of the functional STING pathway in hepatocytes hampers efficient innate control of HBV infection	^105^
	Human immunodeficiency virus (HIV)-1 infection	HIV-1 nonstructural protein can suppress antiviral immunity for immune evasion by targeting STING	^199^
	Influenza A virus (IAV) infection	A STING-dependent, cGAS-independent pathway is important for full interferon production and antiviral control of IAV	^106^
	COVID-19	cGAS–STING signalling is a critical driver of aberrant type I IFN responses in COVID-19	^201^
	Human rhinoviruses infection	Replication of Human rhinoviruse A serotypes is strictly dependent on STING	^114,115^
Bacterial infection	*Mycobacterium tuberculosis* infection	STING deficiency resulted in increased bacterial survival inside macrophages	^299^
	*Brucella abortus* infection	Lack of STING renders macrophages in inefficient to kill Brucella, resulting in an increased bacterial burden	^300^
Protozoan parasites infection	*Plasmodium* infection	Genomic DNA from *Plasmodium falciparum*, as the hemozoin-associated cargo, may access the cytosol due to phagosomal destabilization and triggers the cGAS–STING pathway	^301^
	*Toxoplasma gondii* infection	IRF3-mediated STING signaling is essential for *T. gondii* replication	^302^
	*Leishmania* infection	STING-mediated IFN-β production enhances the intracellular survival of *Leishmania*	^303^
	*Trypanosoma cruzi* infection	STING agonist as the immunological adjuvant protects against infection by different *T. cruzi* strains	^304^
Autoimmune diseases	STING-associated vasculopathy with onset in infancy (SAVI)	Disease caused by several gain-of-function mutations in STING1	^12^
	Aicardi–Goutières syndrome (AGS)	A subset of AGS eticological genes leads to cytosolic nucleic acid accumulatio and cGAS-STING pathway activation	^162^
	Familial chilblain lupus	A heterozygous gain-of-function mutation in STING can cause familial chilblain lupus	^207^
	COPA syndrome	Aberrant activation of the STING pathway due to its deficient retrograde from Golgi to ER	^120–123^
	Niemann–Pick disease type C	Knockout of NPC1 ‘primes’ STING signalling by ‘tethering’ STING to SREBP2 trafficking and blocking STING lysosomal degradation	^97^
	Systemic lupus erythematosus	Subset of patients has elevated cGAMP levels.	^305^
	Rheumatoid arthritis	Reduced cytokine expression in patient cells following cGAS or STING knockdown	^306^
Neurological disorders	Parkinson disease	Inflammatory phenotype in mice model is completely rescued by concurrent loss of STING	^210^
	Huntington’s disease	cGAS promotes the inflammatory and autophagy responses in Huntington’s disease	^212^
	Amyotrophic lateral sclerosis (ALS)	TDP-43 causes inflammation in ALS by stimulating mitochondrial DNA release and cGAS/STING pathway activation	^211^
	Multiple sclerosis	Activation of the STING attenuates experimental autoimmune encephalitis, a model of multiple sclerosis	^307^
	Chronic pain	Mice lacking STING or IFN-I signalling exhibit hypersensitivity to nociceptive stimuli and heightened nociceptor excitability	^209^
	Aautistic-like behaviors	Deficiency of STING signaling in the embryonic cerebral cortex leads to neurogenic abnormalities and autistic-like behaviors	^208^
Cardiovascular diseases	Myocardial infarction	Protection in cGAS-deficient mice or mice receiving STING inhibitor treatment	^213–215^
	Atherosclerosis	Loss of STING reduces atherosclerotic lesions, macrophage accumulation in plaques, and inflammatory molecules in mouse models	^217^
	Aortic aneurysm and dissection (AAD)	The presence of cytosolic DNA and subsequent activation of STING signaling represent a key mechanism in aortic degeneration	^216^
	Cardiac hypertrophy	Genetic or pharmacologic inhibition of the myocardial mitochondria–STING–NF-κB axis prevents chronic kidney disease (CKD)-associated cardiac hypertrophy	^308^
Metabolic diseases	Obesity	cGAS–cGAMP–STING pathway plays an important role in mediating obesity-induced metabolic dysfunction	^164^
	Type 2 diabetes	Global STING knockout beneficially alleviates insulin resistance and glucose intolerance induced by a high-fat diet, but STING knockout in islet cells impairs its glucose-stimulated insulin secretion	^161^
	Nonalcoholic steatohepatitis (NAFLD)	STING-mediated inflammation in Kupffer cells and monocyte-derived macrophages contributes to the progression of NAFLD	^223,309^
Digestive system diseases	Acute pancreatitis	STING senses DNA from dying acinar cells and promotes inflammation in a mouse model of acute pancreatitis	^220^
	Chronic pancreatitis	Unlike acute pancreatitis, STING activation protects chronic pancreatitis by diminishing the generation of IL-17A	^221^
	Inflammatory Colitis	STING knockout mice are highly susceptible to dextran sodium sulfate-induced colitis and T-cell-induced colitis	^219^
Aging	Senescence and aging	Protection against senescence seen in cGAS-deficient or STING-deficient cells or mice	^225,310,311^

*cGAMP* cyclic GMP–AMP, *cGAS* cyclic GMP–AMP synthase, *IRF3* interferon regulatory factor 3, *MLH1* mutL homolog 1, NPC1 NPC intracellular cholesterol transporter 1, *STING* stimulator of interferon genes, *TDP-43* transactive response DNA binding protein 43, *SREBP2* sterol regulatory element binding protein 2.

Interestingly, the tumor cell itself can intrinsically activate the cGAS–STING pathway due to its genome instability.^186^ The rupture of the micronuclear envelope exposes the genome DNA to the cytosol and in some cases mitochondrial dysfunction results in the release of mitochondrial dsDNA.^187^ Such tumor cell-derived cGAMP can be transferred into immune cells through cell gaps. Many newly identified channels mediate this transfer of 2’3’-cGAMP within the tumor environment, including connexins,^188^ connexin 43-PCDH7 gap junctions,^189^ SLC19A1,^190,191^ SLC46A2,^192^ and volume-regulated anion channels (VRAC) LRRC8.^193–195^ Extracellular cGAMP could be redistributed by regulating these transporters, thus being harnessed to treat cancers with low immunogenicity.^196^

In some cases, however, STING can also play pro-tumorigenic roles in the background of chronic inflammation. In contrast to tumor suppression enabled by acute activation of STING, chronic inflammation in the tumor environment provides a promoting niche for carcinogenesis. For example, mutagenic 7,12-dimethylbenz(a)anthracene (DMBA), cisplatin, and etoposide promote skin carcinogenesis by producing STING-dependent inflammatory cytokines and phagocytic infiltration.^197^ Similarly, chromosomally unstable tumor cells co-opt chronic activation of noncanonical NF-κB signalling downstream of STING to promote cell invasion and metastasis.^187^ Brain metastatic cancer cells can hijack the astrocyte STING pathway to maintain the production of IFNα and TNFα, which in turn activate the STAT1 and NF-κB pathways in brain tumor cells, promoting tumor growth and chemoresistance.^189^ Additionally, STING activation can also confer immunosuppression by recruiting myeloid-derived suppressor cells through the CCR2 pathway^197^ or as up-regulating immunosuppressive proteins such as programmed death ligand 1 (PD-L1) and IDO1.^198^ The underlying mechanism dictating these fundamentally contrasting outcomes of STING activation in tumors deserves further investigation.

### STING in infection

Boosting the STING pathway also holds promising applications in combating infection. Since its discovery, multiple animal data supported that STING knock mice are more susceptible to DNA virus infection.^4^ The human and murine hepatocyte has been reported to be devoid of STING expression^199^ and a functional innate DNA-sensing pathway, partially explaining the tropism of the hepatitis B virus towards hepatocytes. Importantly, the introduction of STING expression specifically in hepatocytes leads to improved control of HBV in vivo. However, in addition to hepatocytes, STING has intact expression in cells such as Kupffer cells and resident myeloid cells. In the murine cytomegalovirus infection model, STING is involved in early IFN-β induction in Kupffer cells and the restriction of viral dissemination through myeloid cells.^200^ Although the distinction clearly exits between the RNA and DNA sensing pathways in a cell-based biochemical assay, these distinctions are less clear when actual antiviral activities are examined in physiological contexts. As mentioned above, several studies support that STING also counts in immunity against RNA virus infection. Just in the pandemic of COVID-19, it has been reported that cGAS-STING activity was detected in lung samples and skin lesions from patients infected with SARS-CoV-2. The STING-dependent type I IFN signature is primarily mediated by macrophages and the adjacent endothelial cells with mitochondrial DNA release. And pharmacological inhibition of STING reduces severe lung inflammation induced by SARS-CoV-2 and improves disease outcomes in mouse model.^201^ Controlling aberrant and prolonged type I IFN responses could reduce tissue damage. However, a proper and fine-tuned induction of type I IFNs can also limit virus propagation. The agonists of STING are suggested to be a potential adjuvant due to their ability to enhance antigen-specific antibody production and T-cell responses in mice. To support this, chitosan, a candidate vaccine adjuvant, was shown to exert an increasing effect by activating cGAS–STING signaling and promoting dendritic cell maturation and Th1 cell responses.^202^ And preclinical data confirmed that STING agonist-adjuvanted vaccines generate potent and durable neutralizing antibody and T cell responses.^203^ Furthermore, STING is also involved in the infection of bacterial and protozoan parasites, which is reviewed elsewhere.^204,205^

### STING in autoimmune diseases

Overactivation of STING can cause an undesirable inflammatory response and lead to autoimmune diseases. Several monogenic autoinflammatory syndromes are marked by overactivation of STING due to gain-of-function mutation of STING, abnormal metabolism of nucleic acid metabolism, or forced trafficking of STING. In 2013, several STING1 mutations were reported to be associated with the onset of a severe autoinflammatory syndrome in children named STING-associated vasculopathy with onset in infancy (SAVI), which is characterized by early-onset systemic inflammation, cutaneous vasculopathy, and pulmonary inflammation.^12^

Disturbed self-DNA metabolism caused by a mutation in the TREX1 gene and genes encoding the three RNase H2 endonuclease subunits, RNASEH2A, RNASEH2C, and SAMHD1 is the etiology of another rare genetic disorder, Aicardi–Goutières syndrome.^162^ Similarly, hypomorphic mutations in DNASE2 are related to a clinical syndrome with an elevated type I interferon signature, neonatal anaemia, kidney disease, and arthropathy.^206^ All these presentations in disease animal models can be compromised by depletion of either cGAS or STING. Similarly, familial chilblain lupus is a monogenic form of cutaneous lupus erythematosus caused by loss-of-function mutations in the nucleases TREX1 or SAMHD1. However, in a family without TREX1 or SAMHD1 mutation, heterozygous gain-of-function mutation in STING can encapsulate similar manifestations of familial chilblain lupus.^207^

Forced translocation of STING from the ER to the Golgi apparatus also drives its abnormal activation. COPI is critical for the retrieval of proteins from the Golgi to the ER and for intra-Golgi transport.^120–122^ COPA syndrome is a rare early-onset autosomal dominant disease caused by missense mutations in the COPA gene, which encodes the COP-α protein of the COPI complex, and is characteristic of immune dysregulation with elevated type I interferon signaling.^120^ As COPI-mediated reverse translocation of STING from Golgi back to ER, it is reported that COPA mutations dictate the onset of elevated type I interferon signature by promoting ligand-independent activation of STING-mediated signaling. Interestingly, COPA dysfunction can be reduced by genetic or pharmacological interference with STING.^121^ Niemann-Pick disease type C1 is a rare inherited neurodegenerative disease with a mutation in NPC1, which leads to the accumulation of cholesterol and other lipids in the lysosome, resulting in low levels of cholesterol in the ER and activation of SREBP2-SCAP translocation from the ER to the Golgi. As SREBP2 is another STING interacting protein along translocation, the knockout of Npc1 in the mouse model induces STING activation by physically tethering STING to SREBP2 trafficking.^97^

### STING in neurological disorders

As mentioned above, STING presents versatile outputs and induces divergent responses among different cells. Especially in highly specialized and nonimmune cells, STING as a ubiquitous gene may mediate housekeeping functions. It is reported that a deficiency of STING signaling in the embryonic cerebral cortex leads to neurogenic abnormalities and autistic-like behaviors. In this condition, STING activates nuclear factor κB (NF-κB) to trigger aristaless-like homeobox 4 (ALX4) transcription, which is a key effector in brain development.^208^ Interestingly, type I interferon signaling of STING has also been reported to control nociception in sensory neurons.^209^ Thus, the STING agonist may also alleviate chronic pain, including cancer pain. Furthermore, STING-mediated inflammation is also associated with several neurodegenerative diseases, including Parkinson’s disease and amyotrophic lateral sclerosis (ALS). PARKIN and PINK1, two proteins closely related to Parkinson’s disease, function within the same biochemical pathway and remove damaged mitochondria through selective autophagy, namely mitophagy. Following induction of acute (exhaustive exercise-induced) or chronic (mtDNA mutation-induced) in vivo mitochondrial stress, mice deficient in Parkin or Pink1 accumulate mtDNA and present a type I interferon response in a STING-dependent way.^210^ The cytoplasmic and mitochondrial accumulation of TDP-43 is a hallmark in many cases of ALS and frontotemporal lobar degeneration (FTLD). Mechanically, mislocalized mitochondrial TDP43 causes mtDNA release through mitochondrial permeability transition pore (MPTP) opening and leakage through VDAC1, resulting in the cGAS-STING-dependent induction of type I interferons and inflammatory cytokines.^211^ Expansions of a repeat hexanucleotide (GGGGCC) in the C9orf72 gene are the alternative cause of familial ALS and FTD. Loss of C9orf72 from myeloid cells alone is sufficient to trigger early activation of the type I interferon through impaired degradation of STING. Lastly, the cGAS-STING pathway is also hyperactivated in the Huntington disease model, mediating inflammatory and autophagy responses.^212^

### STING in cardiovascular diseases

**Fig. 7:**
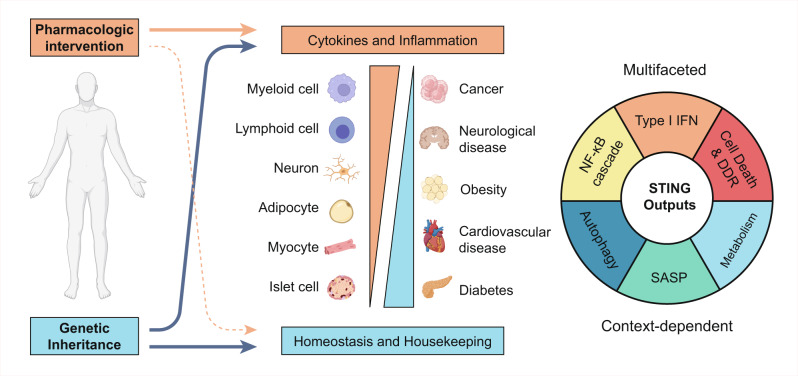
STING involvement in different diseases from a reductionist perspective. STING presents multifaceted outputs in highly differentiated cells. In immune cells like the myeloid line and lymphoid line, STING activation mainly results in cytokine secretion and inflammation induction, while in cells like pancreatic islet cells or myocytes, STING may act as a housekeeping protein to maintain the homeostatic function of cells. In this regard, STING depletion in such cells may impair physiological function. Current pharmacological intervention towards STING mainly targets its cytokine-inducing function and is aimed at inflammation-dominant diseases, such as cancers and auto-immune diseases. However, the genetic knockout of STING in animal models may comprehensively turn off all its outputs, including those in both immune cells and structural cells. Thus, the control of STING in different diseases may yield the opposite outcome or zero-sum phenotypes, depending on the composing proportion of the multifaceted roles of STING. DDR DNA damage response, SASP senescence-associated secretory phenotype. Created with BioRender.com

Sterile inflammation or unresolved chronic inflammation is the characteristic of common cardiovascular diseases, such as myocardial infarction, ischemia-reperfusion injury, atherosclerosis, and aortic aneurysm and dissection (AAD). Upon diversified injury, the release of nucleic acids becomes a general trigger of the cGAS-STING pathway in these diseases. Myocardial infarction (MI) is a disease that involves both cardiomyocyte death and an acute inflammatory response. Ischemic injury activates cGAS-mediated signaling, possibly through the detection and binding of nuclear DNA and mitochondrial DNA released from the necrotic myocardium. Activation of cGAS activation promotes tissue destruction by maintaining pro-inflammatory macrophages, while silencing of cGAS promotes macrophage transformation to a reparative phenotype (like M2) that promotes efficient repair, mitigates adverse remodeling, and improves cardiac function.^213^ The STING inhibitor can produce a beneficial outcome on myocardial infarction.^214^ However, MI survival is not improved in STING null mice, but in mice genetically knockout of IRF3, or the type I IFN receptor IFNAR, and mice with an IFNAR neutralizing antibody.^215^ It suggests that the benefits of restrained inflammation by STING depletion may be offset by the unknown protective function in structural cells that do not secrete IFN. One explanation is that some outputs of STING are independent of the polymerization of STING and cannot be targeted by such inhibitors. But in STING knockout mice, all downstream signals of STING are indiscriminately quenched. Therefore, the function of STING in diseases is the sum of the specific effects in different cell types and varies between different diseases (Fig. 7). Sporadic AAD, caused by progressive loss of aortic smooth muscle cells (SMCs) and degradation of the extracellular matrix, is another highly lethal cardiovascular disease. The presence of cytosolic DNA in SMCs and macrophages and significant activation of the STING pathway is observed in human sporadic AAD tissues. Mechanically, nuclear and mitochondrial DNA damage in SMCs and subsequent leakage of DNA to the cytosol activated STING signaling, which induced cell death through apoptosis and necroptosis.

In addition, DNA from damaged SMCs was engulfed by macrophages which activated STING and its target interferon regulatory factor 3, which directly induced the expression of matrix metalloproteinase-9. STING knockout mice showed significant reductions in challenge-induced aortic enlargement, dissection, and rupture in the thoracic and abdominal aortic regions.^216^ In atherosclerosis models, the diseased aorta showed a higher expression of STING and a higher level of cGAMP. Both genetic deletion and pharmacological blockade of STING improved atherogenesis, lipid and macrophage accumulation in plaques, and inflammatory molecule expression.^217^

### STING in digestive system diseases

The microbiota in the gut serves as a natural source of bacterial cyclic dinucleotide, with a potential link to the STING activation in surrounding tissues. It has been reported that the normal gut microbiota can mediate systemic priming of the cGAS–STING–IFN-I axis through the release of DNA-containing membrane vesicles, protecting distal organs against viral infection in a state of constant preparedness.^218^ STING is also required for intestinal homeostasis. STING knockout mice presented defective protective mechanisms of the intestinal mucosa and were more susceptible to dextran sodium sulfate-induced colitis, T-cell-induced colitis, and enteric Salmonella typhimurium infection.^219^ STING signaling was also activated in the pancreata of mice with acute pancreatitis, while macrophage transfer from STING-knockout mice can ameliorate pancreatic injury and present lower serum levels of lipase and pancreatic trypsin activity.^220^ However, in chronic pancreatitis, STING activation is protective by regulating adaptive immune responses and diminishing the generation of IL-17A. These data also indicated that STING requires differential targeting in different closely related diseases.^221^

### STING in metabolic diseases

Chronic inflammation in adipose tissue plays a key role in obesity-induced insulin resistance. Fat-specific knockout of the DsbA-L, a chaperone-like protein originally identified in the mitochondrial matrix, was reported to impair mitochondrial function and promote mtDNA release, leading to activation of the cGAS–STING pathway and inflammatory responses.^164^ STING levels also increased in the liver tissues of patients with nonalcoholic fatty liver disease (NAFLD) and mice with HFD-induced steatosis.^222^ STING-mediated inflammation in Kupffer cells and macrophages contributes to the progression of NAFLD.^222,223^ A more interesting case is the contrasting phenotype between global STING knockout mice and β-cell-specific STING knockout mice, in the context of the type 2 diabetes (T2D) model. STING knockout mice benefit from insulin resistance and glucose intolerance induced by the high-fat diet, whereas STING-βKO mice present impaired insulin secretion stimulated by islet glucose. In the islet cell, STING fine-tunes the function of the transcription factor Pax6 to maintain normal insulin secretion, which is dampened in the islets of db/db mice and patients with T2D.^161^

### STING and senescence

STING is also closely related to cell senescence and aging, wherein senescent cells lost the capacity to proliferate, impair tissue function and are characteristic of the heightened release of cytokines, chemokines, and proteases to the extracellular milieu, referred to as the senescence-associated secretory phenotype (SASP).^224^ The presence of cytoplasmic chromatin fragments that pinch off from intact nuclei of primary cells is found to emerge during senescence. Such genomic DNA serves as a reservoir to activate cGAS–STING cascading and initiate a chronic inflammation that associates with tissue destruction and cell senescence.^225^ Some of these cytokines could provide a critical paracrine signal back to the secreting cells to sustain cellular senescence. Among them, Type I IFNs promote senescence by inducing DNA damage and elevating the p53 level.^226^ In a cell-intrinsic manner, STING activation can also initiate cell senescence through PERK–eIF2α axis, which is independent of the cytokine induction.^15^ Several mouse models also validate the essential role of STING in senescence and aging. The depletion of TFAM, a well-known trigger of cGAS–STING activity in multiple settings, in T cells is sufficient to trigger an accelerated ageing phenotype and multimorbidity by promoting a pro-senescent inflammatory milieu in vivo.^227^ As radiation-induced genomic damage was previously shown to cause a loss of renewal of melanocyte stem cells. It is postulated that STING-mediated SASP may contribute to regulating these stem cells because STING null mice display remarkably less hair graying months after irradiation.^225^ Taken together, inhibitors of the STING pathway may offer therapeutic effects on senescence and age-related diseases.

## STING-related targeted therapies

Targeting STING with drugs is another worthwhile issue. Such attempts are first tested in the field of oncotherapy. Multiple clinical trials that involve STING agonists are on the go (Table 4). In the preclinical stages, several strategies are operating in parallel to develop STING-targeted modulators. The most straightforward one is to develop nucleotide cGAMP mimetics, which can be applied for the treatment of solid accessible tumors amenable to intratumoral delivery. Most recently, artificial biosynthetic pathways using an engineered kinase-cGAS cascade to produce nucleotide cGAMP mimetic have been established with substantial production capacity.^228^

**Table 4:** Clinical trials about STING agonists

Agent	First posted data	Route of delivery	Co-therapy	Conditions	Status	Phases	Estimated/Actual Enrollment	First Posted Data	NCT Number
GSK3745417	2022/6/21	i.v.	Alone	Leukemia, myeloid, acute	Not yet recruiting	I	72	2022/6/21	NCT05424380
	2019/2/18	i.v.	Alone and combined with dostarlimab	Neoplasms	Recruiting	I	300	2019/2/18	NCT03843359
TAK-500	2021/10/7	i.v.	Alone and combined with a checkpoint inhibitor pembrolizumab	Pancreatic cancer, hepatocellular cancer, squamous cell cancer (SCC), mesothelioma, non-small cell lung cancer (NSCLC), breast cancer, gastric cancer, esophageal cancer	Recruiting	I	106	2021/10/7	NCT05070247
TAK-676	2021/5/10	i.v.	Alone and combined with pembrolizumab following radiotherapy	Carcinoma, non-small-cell lung, triple-negative breast neoplasms, squamous cell carcinoma of head and neck	Recruiting	I	65	2021/5/10	NCT04879849
	2020/6/9	i.v.	Alone and combined with pembrolizumab following radiotherapy	Solid neoplasms	Recruiting	I	288	2020/6/9	NCT04420884
SNX281	2020/10/30	i.v.	Alone and combined with a checkpoint inhibitor Pembrolizumab	Advanced solid tumor, advanced lymphoma	Recruiting	I	134	2020/10/30	NCT04609579
SB 11285	2019/9/20	i.v.	Alone and combined with Atezolizumab	Melanoma and head and neck squamous cell carcinoma and solid tumor	Recruiting	I	110	2019/9/20	NCT04096638
CDK-002	2020/10/19	i.t.	Alone	Advanced solid tumor	Active, not recruiting	I/II	27	2020/10/19	NCT04592484
BI 1387446	2019/11/1	i.t.	Alone and combined with Ezabenlimab	Neoplasms	Recruiting	I	120	2019/11/1	NCT04147234
BMS-986301	2019/5/21	i.m., or i.i. or i.v.	Alone and combined with Nivolumab and Ipilimumab	Advanced solid cancers	Recruiting	I	190	2019/5/21	NCT03956680
E7766	2019/9/30	intravesical injection	Alone	Urinary bladder neoplasms	Withdrawn	I	0	2019/9/30	NCT04109092
	2019/10/30	i.t.	Alone	Lymphoma and advanced solid tumors	Recruiting	I	120	2019/10/30	NCT04144140
IMSA101	2019/7/15	i.t.	Alone and combined with an immune checkpoint inhibitor	Solid tumor, adult	Recruiting	I /II	115	2019/7/15	NCT04020185
MK-1454(Ulevostinag)	2020/1/7	i.t.	Alone and combined with Pembrolizumab (MK-3476)	Head and neck squamous cell carcinoma (HNSCC)	Active, not recruiting	II	200	2020/1/7	NCT04220866
	2017/1/4	i.t.	Alone and combined with Pembrolizumab (MK-3475)	Solid tumors and lymphoma	Completed	I	157	2017/1/4	NCT03010176
SYNB1891	2019/11/18	i.t.	Alone and combined with Atezolizumab	Metastatic solid neoplasm, lymphoma	Recruiting	I	70	2019/11/18	NCT04167137
MK-2118	2017/8/15	i.t. or s.c.	Alone and combined with Pembrolizumab (MK-3475)	Solid tumor and lymphoma	Active, not recruiting	I	160	2017/8/15	NCT03249792
ADU-S100	2019/5/3	i.t.	Alone	Metastatic head and neck cancer, recurrent head and neck cancer	Terminated	II	16	2019/5/3	NCT03937141
	2017/6/1	i.t.	Alone and combined with anti-PD-1 antibody PDR001	Solid tumors and lymphomas	Terminated	I	106	2017/6/1	NCT03172936
	2016/2/5	i.t.	Alone and combined with ipilimumab	Advanced/metastatic solid tumors or lymphomas	Terminated	I	47	2016/2/5	NCT02675439
DMXAA	2009/3/18	i.v.	Alone	Solid tumors	Completed	I	63	2009/3/18	NCT00863733
	2009/3/5	i.v.	Alone	Refractory tumors	Completed	I	15	2009/3/5	NCT00856336
	2009/1/30	n.s.	Alone and combined with carboplatin and paclitaxel	Non-small cell lung cancer	Completed	I /II	105	2009/1/30	NCT00832494
	2004/5/20	i.v.	Alone	Unspecified adult solid tumor, protocol specific	Completed	I	3	2004/5/20	NCT00003697
	2011/1/28	n.s.	Alone	Advanced or recurrent solid tumors	Completed	I	9	2011/1/28	NCT01285453
	2008/4/21	i.v.	Combined with carboplatin and paclitaxel	Non-small cell lung cancer	Terminated	III	1285	2008/4/21	NCT00662597
	2008/8/20	i.v.	Combined with Docetaxel	Non-small cell lung cancer	Terminated	III	900	2008/8/20	NCT00738387
	2011/9/2	n.s.	Combined with Fluvoxamine in core Phase, and combined with either paclitaxel or docetaxel or paclitaxel plus carboplain chemotherapy combination in Extension Phase	Solid tumors	Terminated	I	17	2011/9/2	NCT01299415
	2011/2/7	n.s.	Combined with standard chemotherapy	Solid tumor malignancies	Terminated	I	54	2011/2/7	NCT01290380
	2009/12/14	i.v.	Combined with cetuximab, carboplatin, and paclitaxel	Tumors	Withdrawn	I	0	2009/12/14	NCT01031212
	2010/2/19	i.v.	Combined with docetaxel	Urothelial carcinoma	Withdrawn	II	0	2010/2/19	NCT01071928
	2010/1/27	i.v.	Combined with Carboplatin, Paclitaxel and Vadimezan	Lung cancer	Completed	II	17	2010/1/27	NCT01057342
	2005/5/25	i.v.	Combined with docetaxel	Prostate cancer	Completed	II	70	2005/5/25	NCT00111618
	2011/1/19	n.s.	Alone	Histologically proven and radiologically confirmed solid tumors	Terminated	I	5	2011/1/19	NCT01278849
	2011/1/19	i.v.	Alone	Metastatic cancer	Terminated	I	7	2011/1/19	NCT01278758
	2008/5/7	i.v.	Combined with paclitaxel and carboplatin	Non-small cell lung cancer	Completed	I	15	2008/5/7	NCT00674102
	2011/2/18	i.v.	Alone	Advanced solid tumors	Terminated	I	7	2011/2/18	NCT01299701
	2010/11/15	i.v.	Combined with paclitaxel plus carboplatin or docetaxel	Metastatic cancer with impaired renal function, metastatic cancer with normal renal function	Terminated	I	27	2010/11/15	NCT01240642

*i.v.* intravenous injection, *i.t.* intratumoral injection, *i.m.* intramuscular injection, *s.c.* subcutaneous injection, *n.s.* no statement.

However, such nucleotide mimetics have poor pharmacokinetics because of metabolic instability and membrane impermeability. Ectonucleotide Pyrophosphatase/Phosphodiesterase 1 (ENPP1) was identified with the ability to hydrolyze cGAMP in an extracellular environment, whose breakdown products include the immune suppressor adenosine, further dampening anti-cancer immunity and promoting tumor metastasis.^229^ To overcome this barrier, immunomodulatory nanosystems then provide an effective strategy to deliver such STING agonists,^230^ while stimuli-responsive nanoparticles further help to achieve targeted and controlled drug release depending on the characteristics of the tumor environment and avoid side effects.^231^ It was reported that endosomolytic polymersomes encapsulating cGAMP can increase cGAMP activity by several orders of magnitude via both intravenous and intratumoral administration routes.^232^ It also enhances STING activation in both the tumor and sentinel lymph node and paves the road for enhanced synergy with immune checkpoint inhibitors.^232^

In parallel, the non-nucleotide STING agonist was designed to overcome the poor pharmacokinetics of the nucleotide, including ABZI,^233^ MSA-2,^234,235^, and SR-717.^236^ Among them, MSA-2 and SR717 are amenable to oral administration, a desirable delivery route because of convenience and low cost. Natural compounds with activity to selectively regulate STING may serve as valuable resources for screening. Several natural compounds with the capacity to modulate STING activation were also discovered,^237^ which may serve as the leading compound for further modification. Given the mechanism of selective human STING agonist C53 and the concept of a second pocket, it would be interesting to develop novel modulators targeting the second pockets of STING.

Another strategy to induce STING activation comes from a report about a polyvalent STING agonist—a synthetic polymer with a cyclic seven-membered ring (PC7A)—which binds to a non-competitive STING surface site that is distinct from the cGAMP binding pocket and induces phase condensation of STING.^62,238^ Therefore, it can also effectively induce the activation of the cGAMP-resistant STING variants, such as the natural R232H STING variant. In addition, such polymer-mediated STING biomolecular condensates are more resistant to degradation, generating a delayed and durable STING activation profile. Given separating activation mechanisms, the polymer synergizes with cGAMP to yield the most optimal STING activity profile with a rapid and durable response.^62^

As seen in the clinical trials of STING-based therapies (Table 4), an obvious trend is the combined therapy with immune checkpoint inhibitors, including programmed death-1/ programmed death-ligand 1 (PD-1/PD-L1) blocking antibody and cytotoxic T-lymphocyte antigen 4 (CTLA-4) blocking antibody. Most recent research further proved the efficacy of some novel combination regimes. For instance, a combination of STING agonist and CXCR3 antagonist was reported to overcome anti-PD-L1 resistance in lung adenocarcinoma under oxidative stress.^239^ And a combination of oral STING agonist MSA-2 and anti-TGF-β/PD-L1 bispecific antibody YM101 can effectively overcome immunotherapy resistance in immune-excluded and immune-desert models.^240^ In addition, a methoxy poly(ethylene glycol) (mPEG)-masked CD44×PD-L1/CD3 trispecific T-cell nanoengager was loaded with the STING agonist can transform the cold tumor into a hot tumor and eradicate the large established triple-negative breast cancer.^241^ Mn, an adjuvant to cGAS–STING activation, is also essential for anti-tumor effect, as Mn-insufficient mice had significantly enhanced tumor growth and metastasis and greatly reduced tumor-infiltrating CD8+ T cells.^63,64^ When combined with anti-PD-1 antibody, Mn synergistically boosted antitumor efficacies and reduced the anti-PD-1 antibody dosage required64. Another study designed a thiolated and Mn2+ coordinated cGAMP nanovaccine, which achieve improved control of both the primary and distal tumors.^242^ Lastly, for KRAS-LKB1 mutant lung cancers with STING silenced in epigenetics, it is reported that a single treatment of MPS1 inhibitor can potently re-engage STING activation and restores T cell infiltration through epigenetic de-repression of STING.^243^ Substantial future work is needed to carry forward such a combination into advanced clinical trials.

In addition to the progress of STING agonists, STING inhibitors also hold great potential for treating inflammatory diseases and warrant further investigation in clinical trials. As mentioned above in Table 2, STING underwent substantial post-translational modifications, among which palmitoylation of STING on cysteine residue 91 was essential for STING activation. Ablasser and colleagues identified through a series of compounds as covalent inhibitors of STING, including H-151, and C-170, C-171, C-176, and C-178, which covalently bind to Cys91 in an irreversible way.^244^ Other inhibitors of STING, like Compound 1,^245^ Compound 18,^245^ Astin C,^246^ and SN-011,^247^ are identified to target the ligand-binding pocket of STING via either cell-based phenotypic chemical screen or in silico docking screen. Such inhibitors can attenuate STING-associated autoinflammatory disease in mice^233,246,247^ and provide proof of concept that STING antagonists are efficacious in the treatment of autoinflammatory diseases.

## Conclusions and perspectives

In this review, we systemically review the current knowledge on STING biology in response to activation by cGAMP, and summarize their roles in various diseases as well as STING-related targeted therapies. Since the initial report of STING in 2008, we have learned a lot about the molecule structural information, function, modulation and spatiotemporal distribution of STING. These remarkable achievements benefit from two important technologies, cryo-EM and CRISPR-Cas9 editing. The crystallization of STING, the smallest transmembrane protein to be resolved yet, greatly complements the biochemical experiments and strengthens our understanding of the molecule’s structural information in response to activation. Equipped with CRISPR-Cas9 technology, many regulators involved in STING activation are revealed on a large genome-scale (Table 1). Meanwhile, STING as a primordial protein is discovered to be endowed with versatile biological functionality, which goes beyond cytokine induction, such as autophagy, metabolism regulation, senescence, cell death, DNA damage response, and RNA replication restriction. The in-depth STING interactome and mechanisms of versatile outputs deserve further research in the future.

STING exhibits a crucial role in health and disease for their widespread involvement in various cellular processes (Table 3) (Fig. 7). Particularly in infection, the protective role of STING against invading pathogens, such as DNA viruses, RNA viruses, bacteria and protozoan parasites, is observed both in vivo and in vitro. A strong evidence would come from the report that STING is involved in antiviral immunity in patients with COVID-19.^112^ It has been reported that a STING agonist can act as an adjuvant and induces highly potent and durable neutralizing antibody responses in non-human primates against SARS-CoV-2, suggesting that STING activation may represent a promising therapeutic strategy to control SARS-CoV-2.^203^ However, Ablasser’s group pointed that pharmacological inhibition of STING reduces severe lung inflammation induced by SARS-CoV-2 and improves disease outcome.^201^ These apparently contradictory findings may be explained by differences in the severity of disease and drug administration time. Another important clinical application of STING is to serve as an immunological enhancement in cancer immunotherapy. Mounting evidence implicates that STING activation in the tumor microenvironment elicits a significant tumor regression mediated by the potent antitumor immune response.^234,236,248^ Indeed, many agonists of STING have been tested in clinical trials for cancer immunotherapy. These attempts promote the optimization of dose usage, delivery, and combination regime and also drive the innovation and development of a novel drug with high targeting efficiency but a low side effect. Antagonists of STING also holds promising prospect to treat autoimmune disease, and such clinical trials are less launched and expected to boom in the future.

Given the versatile and context-dependent functions of STING, contrasting therapeutic effects and even side effects may be presented when STING is indiscriminately targeted across different cells. For instance, STING knockout mice manifest improved metabolic parameters exhibited by decreased body weight and reduced insulin resistance, while islet cell-specific STING knockout mice present an impaired islet glucose-stimulated insulin secretion (GSIS), suggesting that STING is required for normal β-cell function.^161^ Therefore, selectively harnessing the outputs of STING in different pathological backgrounds would yield more beneficial outcomes. Based on current understanding, it has been achieved to selectively dampen the IFN induction of STING activation by mutating the key residue on STING CTT essential for IRF3 recruitment, leaving the intact function of NF-κB activation and autophagy induction. Thus, whether other outputs of STING like NF-kB activation, autophagy, and PERK–eIF2α pathway can be modulated selectively is worthy of exploration in the future. Furthermore, developing drugs selectively targeting these outputs is anticipated to achieve better application in clinical practice. Admittedly, these proof-of-concept ideas require an in-depth understanding of the molecular mechanism of these outputs, which still needs intensive exploration in the future. Finally, accurately mapping the cell and tissue type-specific functions of STING in both steady and diseased conditions would help greatly to guide the precise targeting of STING.

Although considerable progress has been made, many open questions are imperative to answer.

1. STING activation is marked with a 180° rotation of the LBD relative to the TM. Such a rotation of STING converts the two connectors that link the LBD and TMD in the STING dimer from the crossover to the parallel configuration. This is quite an interesting molecular event, not found in the activation process of innate immune adaptors like MAVS and TLRs, but evolutionarily conserved feature of both prokaryotic and metazoan STING activation.^33,249^ It is postulated that such a complex process with substantial entropy change must imply underlying biological benefits, which are not well explained.

2. Why and how does STING translocate from ER to Golgi apparatus for activation? The differential membrane composition and luminal biochemical properties of ER and Golgi may be the driving force. Some exclusive factors in the Golgi apparatus may favor STING polymerization and activation. Supporting this view is the discovery of sGASs in the lumen of the Golgi apparatus that binds to the STING transmembrane domain and facilitates its activation.^60^ Or, the ER environment disfavors STING activation, such an oxidizing nature for the formation of disulfide bonds and the high concentration of Ca2^+^ that functions as a folding buffer and is essential for chaperone function.^250^

Currently, no signal peptide for subcellular location is found in STING, and the detailed mechanism by which STING exits the ER is also elusive. COPII is reported to be involved in STING translocation. Recent progress on COPII indicated that hydrophobic mismatching between the transmembrane domains of cargo proteins and the surrounding lipids is essential for cargo sorting.^129^ If STING is a cargo of the COPII system, one speculation is that undergoes conformational changes STING TMD in the process of activation would induce hydrophobic mismatching. However, interacting proteins of STING that are orchestrated in this process need further clarification.

3. It is not clear what an extended human STING filament assembly will be like. STING dimers are packed side-by-side in an approximately linear arrangement to constitute the high-order oligomers of STING. The most recently resolved human STING oligomer only contains four dimers with a curved overall shape,^58^ cryo-EM structure of prokaryotic STING assembly has reached >300 nm in length (about 85 dimer copies, about 6.3 MDa).^249^ Although there exist some technical barriers, it is anticipated to resolve more extended human STING oligomers and reveal the underlying molecular basis of human STING filament extension in the future. And whether the alternative assembly mode of STING exits, possibly in the puzzle-like STING condensate, is also worthy to be studied.

4. How is TBK1 recruited to STING oligomers? In the previously proposed ‘Release of autoinhibition’ model,^57^ TBK1 is believed to be recruited to STING oligomers through the release of the STING CTT, which in steady state is sequestered to the main-body of STING LBD and inaccessible to TBK1 and IRF3. As the CTT of STING is invisible in all current structures, this model needed to be further validated. In addition, although multiple adaptors are confirmed by biochemical assays to involve in STING–TBK1 interaction, these data are not supported by the structural studies. Up to now, the full STING–TBK1 complex is actually a reconstructed model, achieved by rigid-body docking of the structures of human TBK1 with chicken STING.^67^ Thus, future work is needed to understand how TBK1 is recruited to STING oligomers and re-evaluate the necessity of such adaptors in STING–TBK1 interactions, eventually proposing a more detailed model of STING activation.

The ten-year anniversary of the discovery of cGAMP and its synthase cGAS is approaching. Answering these questions and beyond is bound to greatly extend our understanding of comprehensive STING biology, which would also guide a more specific targeting of STING and eventually benefit the clinical practice in relation to STING-related diseases.
